# MrkH, a Novel c-di-GMP-Dependent Transcriptional Activator, Controls *Klebsiella pneumoniae* Biofilm Formation by Regulating Type 3 Fimbriae Expression

**DOI:** 10.1371/journal.ppat.1002204

**Published:** 2011-08-25

**Authors:** Jonathan J. Wilksch, Ji Yang, Abigail Clements, Jacinta L. Gabbe, Kirsty R. Short, Hanwei Cao, Rosalia Cavaliere, Catherine E. James, Cynthia B. Whitchurch, Mark A. Schembri, Mary L. C. Chuah, Zhao-Xun Liang, Odilia L. Wijburg, Adam W. Jenney, Trevor Lithgow, Richard A. Strugnell

**Affiliations:** 1 Department of Microbiology and Immunology, The University of Melbourne, Parkville, Victoria, Australia; 2 The ithree Institute, University of Technology Sydney, Ultimo, New South Wales, Australia; 3 Centre for Infectious Disease Research, School of Chemistry and Molecular Biosciences, The University of Queensland, Brisbane, Queensland, Australia; 4 Division of Chemical Biology and Biotechnology, School of Biological Sciences, Nanyang Technological University, Singapore, Singapore; 5 Department of Biochemistry and Molecular Biology, Monash University, Clayton, Victoria, Australia; Dartmouth Medical School, United States of America

## Abstract

*Klebsiella pneumoniae* causes significant morbidity and mortality worldwide, particularly amongst hospitalized individuals. The principle mechanism for pathogenesis in hospital environments involves the formation of biofilms, primarily on implanted medical devices. In this study, we constructed a transposon mutant library in a clinical isolate, *K. pneumoniae* AJ218, to identify the genes and pathways implicated in biofilm formation. Three mutants severely defective in biofilm formation contained insertions within the *mrkABCDF* genes encoding the main structural subunit and assembly machinery for type 3 fimbriae. Two other mutants carried insertions within the *yfiN* and *mrkJ* genes, which encode GGDEF domain- and EAL domain-containing c-di-GMP turnover enzymes, respectively. The remaining two isolates contained insertions that inactivated the *mrkH* and *mrkI* genes, which encode for novel proteins with a c-di-GMP-binding PilZ domain and a LuxR-type transcriptional regulator, respectively. Biochemical and functional assays indicated that the effects of these factors on biofilm formation accompany concomitant changes in type 3 fimbriae expression. We mapped the transcriptional start site of *mrkA*, demonstrated that MrkH directly activates transcription of the *mrkA* promoter and showed that MrkH binds strongly to the *mrkA* regulatory region only in the presence of c-di-GMP. Furthermore, a point mutation in the putative c-di-GMP-binding domain of MrkH completely abolished its function as a transcriptional activator. *In vivo* analysis of the *yfiN* and *mrkJ* genes strongly indicated their c-di-GMP-specific function as diguanylate cyclase and phosphodiesterase, respectively. In addition, *in vitro* assays showed that purified MrkJ protein has strong c-di-GMP phosphodiesterase activity. These results demonstrate for the first time that c-di-GMP can function as an effector to stimulate the activity of a transcriptional activator, and explain how type 3 fimbriae expression is coordinated with other gene expression programs in *K. pneumoniae* to promote biofilm formation to implanted medical devices.

## Author Summary

Biofilms are surface-associated communities of microorganisms. Biofilm-associated bacteria are protected from host defenses and antibiotics and are the cause of many infections. *Klebsiella pneumoniae* is primarily a hospital-acquired bacterial pathogen that causes pneumonia, urinary tract infections and septicemia. Its success is related to its ability to form biofilms on medical devices, such as catheters. In *K. pneumoniae*, biofilm formation is mediated by type 3 fimbriae – hair-like, protein appendages extending out from the cell surface that adhere to surfaces. This study investigated how *K. pneumoniae* regulates the expression of these fimbriae. We identified a protein, MrkH, which behaves as a “biofilm switch” that turns on the expression of genes responsible for producing type 3 fimbriae. MrkH works by binding to regulatory regions of DNA nearby to these genes and initiates their expression. Importantly, MrkH binds to DNA strongly only when the protein is stimulated by a small molecule, c-di-GMP. Furthermore, we identified bacterial enzymes that either produce or break down c-di-GMP to control its concentration within the cell, and thus modulate MrkH activity. Understanding the molecular basis for these processes may lead to the development of therapeutic compounds, possibly for incorporation into medical device materials to inhibit biofilm formation and pathogenesis.

## Introduction

In the first half of the 20^th^ century, *Klebsiella pneumoniae* was recognized as a community-acquired pulmonary pathogen, chiefly among patients with a history of chronic alcoholism [1]. However, with the advent of more intensive hospital care and the increasingly widespread use of antibiotics, *K. pneumoniae* has become a significant cause of nosocomially-acquired infections among immunocompromised patients, estimated to cause 8% of all nosocomial infections [2], [3], [4], [5], [6], [7]. These infections commonly include pneumonia, urinary tract infection, septicemia and surgical wound infection.

The pathogenesis of nosocomial *K. pneumoniae* infections has been associated with its capacity to form biofilms, particularly on medical devices. *K. pneumoniae* biofilm development is primarily mediated by the mannose-resistant *Klebsiella*-like (MR/K) hemagglutinins or “Mrk proteins” [8]. The Mrk proteins are encoded by an operon which comprises the genes *mrkABCDF* [9]. The Mrk proteins form type 3 fimbriae, cell surface structures that can extend into long filaments (up to 2 µm in length) that attach to surfaces [8], [9], [10], [11], [12]. Type 3 fimbriae are synthesized by the chaperone-usher pathway of protein translocation [13]. All chaperone-usher systems are composed of at least three components: (i) a major pilin subunit, polymerized to form the fimbrial shaft, (ii) a chaperone that folds the pilin subunit in the periplasm to ready it for assembly into the fimbriae and (iii) the usher, a transmembrane β-barrel in the outer membrane that polymerizes the pilin subunit into the growing fimbriae, and serves to extrude this growing fimbriae pilin-by-pilin. In *Klebsiella*, MrkA is the major fimbrial subunit [11], [14], [15] and MrkB and MrkC have the sequence features to represent the periplasmic chaperone and the usher translocase, respectively. The majority of chaperone-usher systems also consist of a fimbria tip-associated adhesion subunit. The MrkD tip adhesion of *K. pneumoniae* is required for mediating adherence to extracellular matrix proteins such as type V collagen, as well as the basement membrane regions and basolateral surfaces of both renal tract and pulmonary epithelia [10], [12], [14], [16], [17].

An additional complexity for adherence and biofilm formation in *K. pneumoniae* is the presence of a thick capsule of polysaccharide surrounding the cells. The capsule is important for biofilm formation, since loss-of-function mutations in the *cps* genes responsible for capsule biosynthesis alter biofilm formation [18], [19]. A previous study, focused on type 1 fimbriae, showed convincingly that fimbria function is inhibited by the presence of the capsule, suggesting direct physical interference with the extension of fimbriae through this 0.5 µm thick, viscous capsule [19]. While it has not been studied, it seems likely that the capsule of *K. pneumoniae* would also impede extrusion of MR/K hemagglutinins.

As part of a large seroepidemiology study of *K. pneumoniae* in Australian hospital settings, our laboratory isolated K54-serotype strains at high frequency [20]. These isolates displayed a high degree of clonality, suggesting a common, nosocomial source [20]. The K54 strain AJ218 was found to be significantly more adherent to urinary catheter material and HEp-2 cells than other clinical isolates [20]. These attributes suggest *K. pneumoniae* AJ218 readily forms biofilms, and may account for the high proportion of infections witnessed in the hospital environment by K54-serotyped strains. We therefore sought to investigate the regulation of biofilm formation in *K. pneumoniae* AJ218.

Here we report the discovery of a ‘biofilm switch’ controlled by a c-di-GMP-binding protein called MrkH. Our data show that the c-di-GMP-dependent switch between the planktonic and biofilm modes of growth is mediated through control of type 3 fimbriae expression. Both *in vivo* and *in vitro* analyses demonstrate that MrkH is a novel PilZ-domain-containing transcriptional regulator, which in the presence of c-di-GMP, activates the expression of the *mrkABCDF* operon by binding to the region immediately upstream of the *mrkA* promoter. We also demonstrate that *K. pneumoniae* encodes a highly active phosphodiesterase (MrkJ) and diguanylate cyclase (YfiN) which appear to contribute to MrkH activity by coordinating cellular c-di-GMP concentration. Together, these results explain how type 3 fimbriae expression in *K. pneumoniae* is regulated in response to factors signaling for biofilm formation.

## Results

### Screening of *K. pneumoniae* transposon mutants for alterations in biofilm formation

To identify factors contributing to rapid biofilm formation by *K. pneumoniae* AJ218, a 7,000 transposon mutant library was constructed and screened in a polyvinyl-chloride (PVC) microtiter plate assay where biofilm formation was quantified by crystal violet staining [21]. Mutants exhibiting reduced or enhanced biofilm ability greater than 15% of *K. pneumoniae* AJ218 were then examined individually.

**Figure 1:**
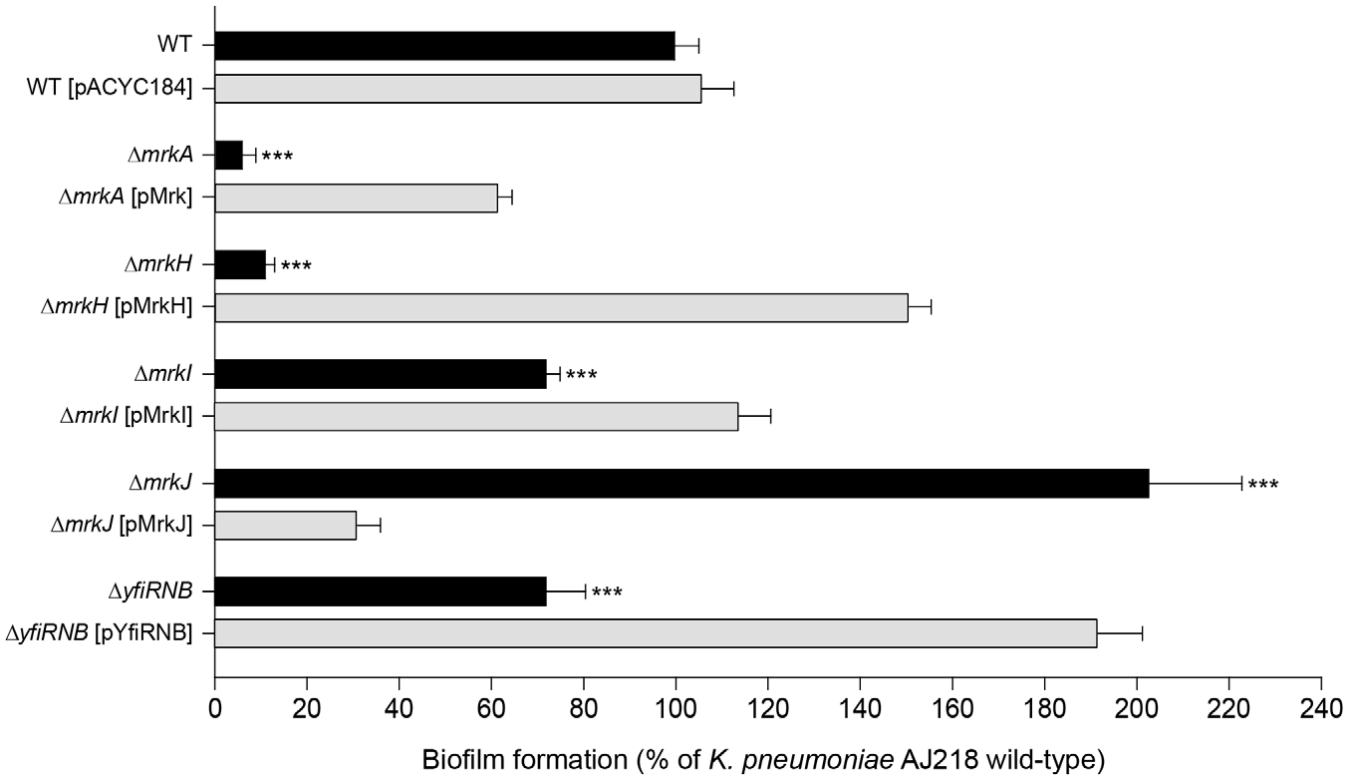
Biofilm formation by *K. pneumoniae* AJ218. Biofilm formation by *K. pneumoniae* AJ218 wild-type, including isogenic mutants and strains harboring trans-complementing plasmids. Biofilm formation was determined using the static microtiter plate assay following incubation in M63B1-GCAA minimal media (supplemented with 1% glycerol and 0.3% casamino acids) for 24 h under static conditions. Results are expressed as a percentage of the biofilm produced by the wild-type AJ218 strain, which is set to 100%. All values represent the mean of four replicate sample wells for each strain performed in two independent experiments. The error bars represent the standard deviation. Statistical significance between AJ218 wild-type and isogenic mutants was analyzed by one-way ANOVA and Tukey HSD post-hoc comparisons are reported, where ***  =  *P*<0.001.

Fifteen biofilm-altered mutants were isolated and the nucleotide sequence immediately flanking each transposon insertion site was identified by Y-linker ligation PCR [22] and sequenced to identify the disrupted locus. Seven mutants were subsequently selected for detailed investigation (Table 1), based upon differences observed in their ability to express functional type 3 fimbriae (see below). The full-length open reading frame (ORF) of each gene disrupted by the transposon insertion was sequenced and exhibited greater than 99% nucleotide identity to homologs located in the sequenced *K. pneumoniae* KTUH-K2022 genome. To confirm the transposon mutant phenotypes, five deletion mutant strains were constructed from wild-type *K. pneumoniae* AJ218 whereby the *mrkA*, *mrkH*, *mrkI*, *mrkJ and yfiRNB* loci were deleted and replaced with a kanamycin resistance-encoding gene. The Δ*yfiRNB* operon deletion was made to compare to the *yfiN* transposon mutant, and to analyze mutants that lacked the entire tripartite signalling module. The kanamycin resistance-encoding gene was excised from the Δ*mrkH* mutant to avoid polar effects on *mrkI* transcription. Each deletion mutant strain exhibited an equivalent defect in biofilm-formation to the initial transposon mutant (Figure 1). No apparent differences in the planktonic growth rates between wild-type, transposon mutant and deletion mutant strains were observed (data not shown). In complementation analysis, empty vector controls had no impact on biofilm formation for all strains tested (Figure S1).

**Table 1:** Identification of genetic loci participating in *K. pneumoniae* AJ218 biofilm formation.

Strain	Gene name/Locus tag^a^	Predicted protein function and characteristics	Predicted ORF (bp) and protein (aa) length	Biofilm formation of Tn*5* mutant (% AJ218^Rif^)^b^
JW30A8	***mrkA***	**Major pilin subunit (MrkA) of type 3 fimbriae**	609 bp; 202 aa	8.9±2.2
	KP1_4561	Main structural component		
JW69A4	***mrkB***	**Chaperone protein (MrkB) for type 3 fimbriae**	702 bp; 233 aa	12.7±4.8
	KP1_4560	Assists subunit folding in the periplasm		
JW8E7	***mrkC***	**Usher protein (MrkC) for type 3 fimbriae**	2487 bp; 828 aa	13.7±8.5
	KP1_4558	Promotes polymerization of pilin subunits		
JW34H5	***mrkH***	**C-di-GMP-binding protein (MrkH)**	705 bp; 234 aa	15.3±6.5
	KP1_4551	Contains PilZ domain		
JW64B4	***mrkI***	**Transcriptional regulator (MrkI)**	594 bp; 197 aa	80.5±7.7
	KP1_4552	LuxR superfamily		
		Contains HTH DNA-binding motif		
JW45H9	***mrkJ***	**Phosphodiesterase (MrkJ)**	717 bp; 238 aa	248.7±14.4
	KP1_4554	Hydrolyzes c-di-GMP		
		Contains EAL domain		
JW34D8	***yfiN***	**Diguanylate cyclase (YfiN)**	1224 bp; 407 aa	86.9±6.1
	KP1_4180	Synthesizes c-di-GMP		
		Contains GGDEF domain		

a Identification of transposon insertion sites in *K. pneumoniae* AJ218 were derived from homology searches of the *K. pneumoniae* NTUH-K2044 genome sequence (GenBank Ref: AP006725).

b Expressed as a percentage of the biofilm produced by *K. pneumoniae* AJ218^Rif^, set to 100%. Determined using the static microtiter plate assay following incubation in M63B1-GCAA (supplemented with 1% glycerol and 0.3% casamino acids) minimal media for 24 h at 37°C. Values represent the mean ± standard deviation, of four replicate sample wells for each strain performed in two independent experiments.

### Type 3 fimbriae of *K. pneumoniae* are an important mediator of biofilm formation

**Figure 2:**
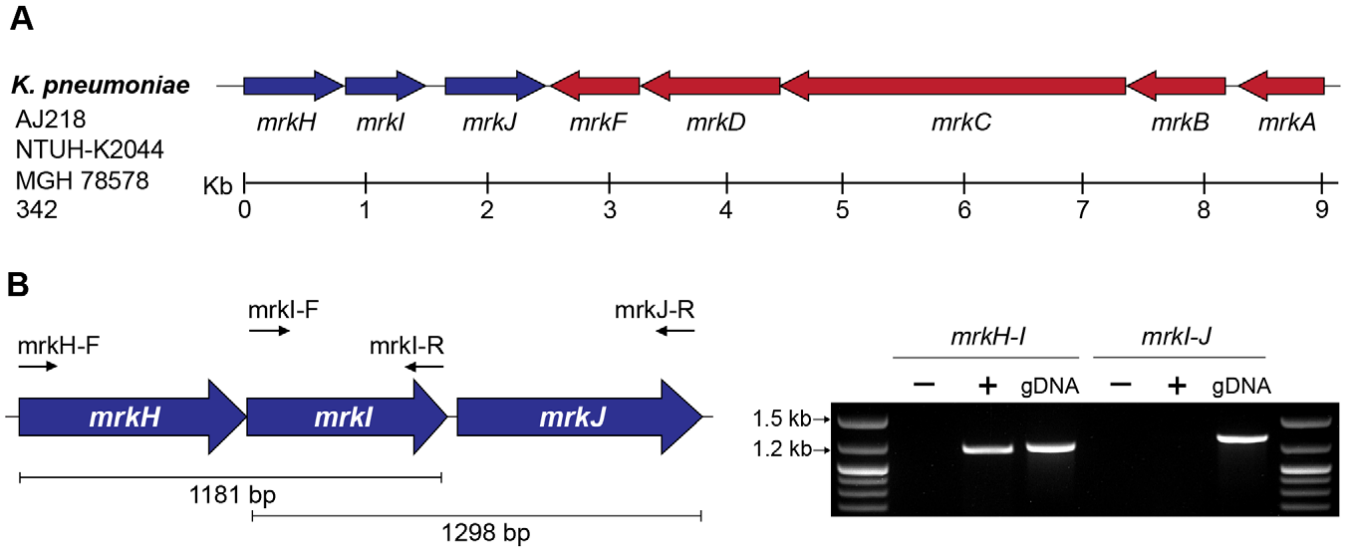
The *mrkABCDF* and *mrkHIJ* loci in *K. pneumoniae* AJ218. (**A**) Genetic organization of the *mrkABCDF* and *mrkHIJ* gene clusters from *K. pneumoniae* strains AJ218, NTUH-K2044 (GenBank Ref: AP006725), MGH 78578 (GenBank Ref: CP000647) and 342 (GenBank Ref: CP000964). (**B**) RT-PCR analysis of *mrkHIJ* transcription. PCR amplicon products of either RNA (−), reverse transcribed DNA (+) or genomic DNA (gDNA) were visualized on a 1% agarose gel. The *mrkH-I* product was generated with primer mrkI-R and amplified with primers mrkH-F and mrkI-R. The *mrkI-J* product was generated with primer mrkJ-R and amplified with primers mrkI-F and mrkJ-R.

The *mrkA* and *mrkB* genes encode the major pilin subunit and periplasmic chaperone of type 3 fimbriae. The *mrkC* gene encodes a protein of 828 amino acids, predicted to have the three domains defining the usher translocase, where residues 156-651 conform to the central translocase domain as defined by Pfam00577 [23]. In contrast to the *K. pneumoniae* AJ218 parent strain, any one of the three mutants: Δ*mrkA,* Δ*mrkB* or Δ*mrkC*, failed to form biofilms (Table 1) and promote MR/K hemagglutination, indicating loss of type 3 fimbriae expression. Complete sequencing of the *mrk* gene cluster of *K. pneumoniae* AJ218 showed five ORFs (*mrkABCDF*) arranged in the same transcriptional orientation (Figure 2A), consistent with other *K. pneumoniae* strains deposited in GenBank.

### The *mrkH*, *mrkI*, *mrkJ* and *yfiRNB* loci contribute to *K. pneumoniae* biofilm formation

Three other mutants isolated from the screen defined a three-locus cluster (*mrkH*- *mrkI*-*mrkJ*) located immediately downstream and transcribed convergently to the *mrkABCDF* operon (Figure 2A and Table 1). The 9 kb region containing the *mrkABCDF* and *mrkHIJ* clusters is highly conserved amongst the sequenced *K. pneumoniae* genomes, with the nucleotide identity between all strains greater than 99%. Analysis of completed bacterial genome sequences showed that the only other species displaying conserved homologs of these two *mrk* clusters is *Citrobacter koseri*, an opportunistic pathogen [24]. Amino acid sequence identities between the Mrk proteins of *K. pneumoniae* and *C. koseri* BAA-895 range from 82% (MrkD) to 92% (MrkB), while homology between the *mrkHIJ* clusters is lower (MrkH = 33%, MrkI = 44%, MrkJ = 73%).

RT-PCR analysis of *K. pneumoniae* AJ218 cDNA templates spanning intergenic regions between *mrkH* and *mrkI* were obtained, but could not be obtained between *mrkI* and *mrkJ* (Figure 2B). These results suggest that the *mrkH* and *mrkI* genes are co-transcribed in a polycistronic mRNA and *mrkJ* is transcribed independently of the *mrkHI* operon.

**Figure 3:**
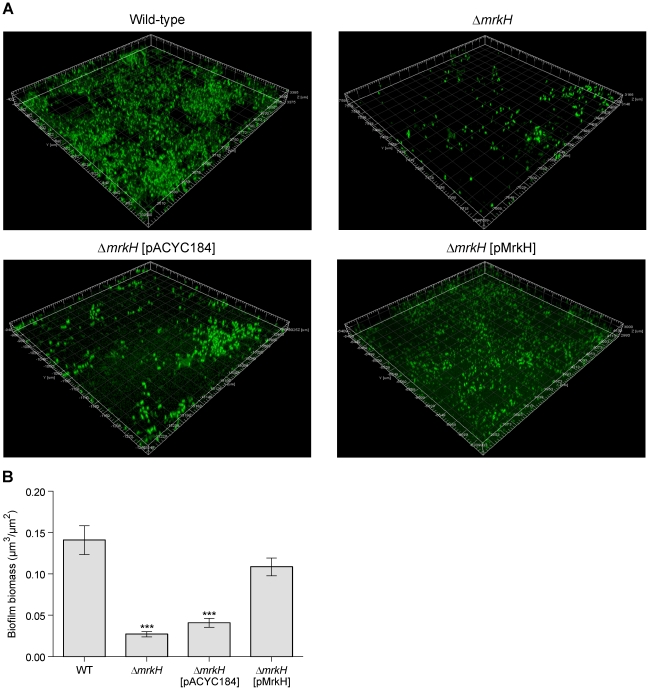
Flow-cell-cultivated biofilm formation by *K. pneumoniae* AJ218 after 4 days. (**A**) Confocal laser scanning micrographs of biofilms formed by *K. pneumoniae* AJ218 wild-type, Δ*mrkH*, Δ*mrkH* [pACYC184] and Δ*mrkH* [pMrkH] strains. Biofilms were stained with Syto64 to visualize cells and are shown in green as maximum intensity volume rendered projections. (**B**) COMSTAT analysis of biofilm biomass. The error bars represent the standard deviation. Statistical significance between AJ218 wild-type and Δ*mrkH* strains was analyzed by one-way ANOVA and Tukey HSD post-hoc comparisons are reported, where ***  =  *P*<0.001.

**Figure 4:**
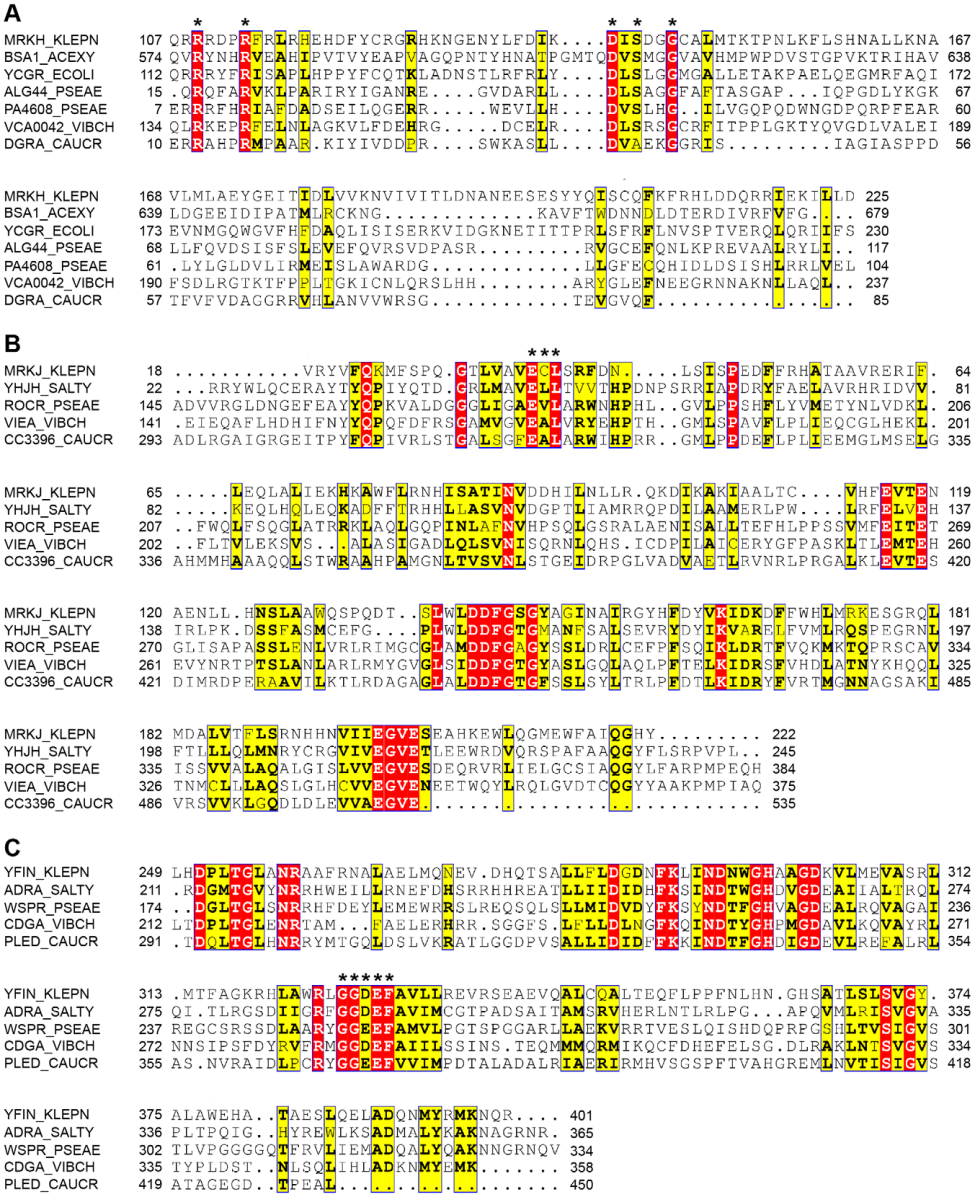
Conservation of PilZ-, EAL- and GGDEF-domain proteins in *K. pneumoniae*. Multiple sequence alignment of the (**A**) PilZ domain of MrkH, (**B**) EAL domain of MrkJ and (**C**) GGDEF domain of YfiN from *K. pneumoniae* AJ218 and other experimentally studied proteins, generated by ClustalW2 [92] and formatted with ESPript [93]. Residues showing strict identity are written in white characters and highlighted in red. Similarity across groups is indicated with black bold characters and highlighted in yellow. Residues required for c-di-GMP binding in the PilZ domain which form two conserved motifs, including the putative catalytic active site residues within the EAL and GGDEF domain, are marked with an asterisk. Protein names and organisms are as follows: MrkH, MrkJ, YfiN: *K. pneumoniae* AJ218; BcsA: *Gluconacetobacter xylinus* NBRC 3288, YcgR: *E. coli* K-12; YhjH, AdrA: *Salmonella* Typhimurium LT2; Alg44, PA4608, RocR, WspR: *Pseudomonas aeruginosa* PA14; VCA0042, VieA, CdgA: *Vibrio cholerae* O395; DgrA, CC3396, PleD: *Caulobacter cresentus* CB15.

The Δ*mrkH* mutant was significantly reduced in biofilm formation, both in a static assay that measures initial biofilm formation (Figure 1) and a continuous flow-cell assay that measures mature biofilm development (Figure 3). For both assays, the mutation could be successfully complemented with wild-type *mrkH* copies (Δ*mrkH* [pMrkH]). The product of *mrkH* contains a putative C-terminal PilZ domain capable of binding the second messenger c-di-GMP. Multiple alignments of the PilZ domain of *K. pneumoniae* MrkH with other experimentally characterized PilZ domain-containing proteins demonstrated complete conservation of two functionally important sequence motifs in MrkH (Figure 4A). The amino acid residues in these conserved motifs (^109^RxxxR and ^140^D/NxSxxG) are known to be crucial for c-di-GMP binding and allosteric regulation of PilZ domain-containing proteins for subsequent downstream functions. We therefore propose that MrkH functions as a c-di-GMP-binding protein.

Biofilm formation by the Δ*mrkI* mutant was moderately deficient compared to wild-type, and could be rescued when supplied with wild-type *mrkI* copies (Δ*mrkI* [pMrkI]; Figure 1). The *mrkI* gene encodes a putative LuxR-like transcriptional regulator that contains a C-terminal helix-turn-helix (HTH) DNA-binding motif. Proteins with LuxR domains can participate in quorum sensing [25], however MrkI lacks the entire N-terminal autoinducer-domain required for binding N-acyl homoserine lactones, suggesting MrkI is a transcriptional activator of gene(s) responding to signals other than those involved in quorum sensing.

The final gene in the three-locus cluster, *mrkJ*, encodes a putative phosphodiesterase (PDE). This enzyme contains an EAL domain that mediates the hydrolysis of c-di-GMP. Multiple alignments of the EAL domain of MrkJ with other known PDE enzymes demonstrated the conservation of several regions throughout the domain sequence (Figure 4B). Like other enzymes of the same class, the residues ‘ECL’ that form the putative active site of MrkJ varies from the consensus ‘EAL’ sequence. The negative regulatory role of EAL-domain proteins over c-di-GMP levels is consistent with the phenotype of both the Δ*mrkJ* mutant (enhanced biofilm) and complemented mutant, Δ*mrkJ* [pMrkJ] (defective biofilm) (Figure 1). This model for biofilm formation is further supported by the identity of the seventh mutant identified in the transposon mutant library screen of *K. pneumoniae* AJ218 (Table 1). The *yfiN* gene encodes a putative integral-membrane diguanylate cyclase (DGC) with a GGDEF domain, which functions to synthesize c-di-GMP. Multiple alignment of the GGDEF domain of YfiN with other studied DGC proteins showed conservation of several regions, including the GGDEF residues that form the putative catalytic active site (Figure 4C). Deletion of the entire *yfiRNB* gene cluster resulted in a significant impairment in biofilm formation and complementation with the *yfiRNB* operon (Δ*yfiRNB* [pYfiRNB]) enhanced biofilm formation to levels almost two-fold greater than wild-type (Figure 1).

We therefore propose that biofilm formation in *K. pneumoniae* is regulated by the relative availability of intracellular c-di-GMP, coordinated by c-di-GMP turnover enzymes (YfiN and MrkJ), and sensed by a receptor protein (MrkH). When a PDE is inactivated or a DGC up-regulated leading to increased c-di-GMP availability, biofilm formation is stimulated. Conversely, when a DGC is inactivated or a PDE up-regulated resulting in decreased c-di-GMP concentration, biofilm formation is inhibited.

### MrkH positively regulates type 3 fimbriae expression in *K. pneumoniae*

**Figure 5:**
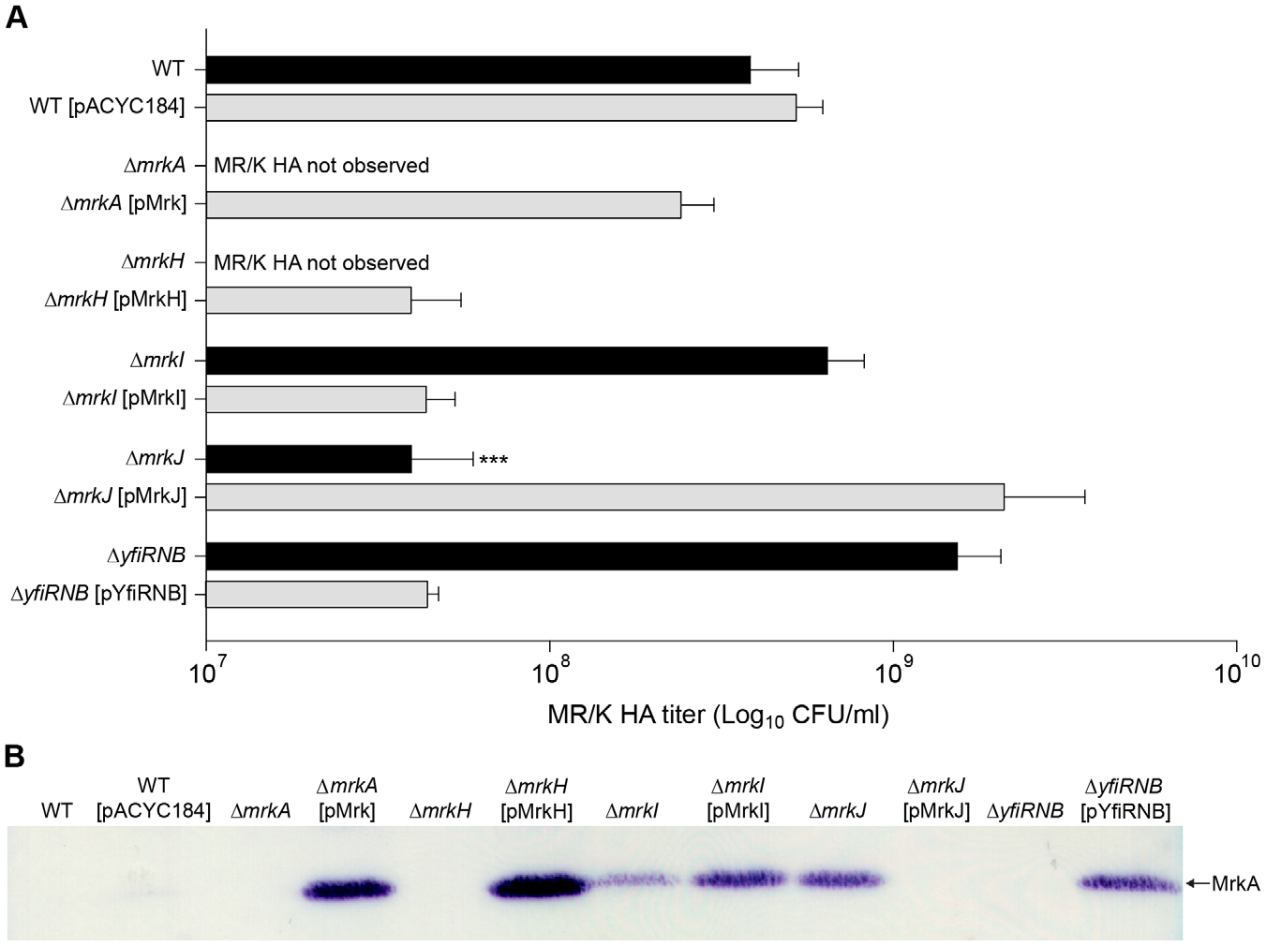
Type 3 fimbriae expression by *K. pneumoniae* AJ218. (**A**) Mannose resistant *Klebsiella*-like hemagglutination (MR/K HA) assays using human erythrocytes. MR/K HA titer is expressed as the lowest concentration (CFU/mL) of bacteria causing a visible agglutination reaction. Values represent the mean of three independent experiments. The error bars represent the standard deviation. Statistical significance between AJ218 wild-type and isogenic mutants was analyzed by one-way ANOVA and Tukey HSD post-hoc comparisons are reported, where ***  =  *P*<0.001. (**B**) Cell lysates were prepared from the indicated strains and analyzed by SDS-PAGE and immunoblotting with anti-MrkA antiserum. The MrkA pilin monomer (which migrates at approximately 21 kDa) is labeled.

We sought to address whether the activity of MrkH influences type 3 fimbriae expression. A sensitive functional assay for type 3 fimbriae is the MR/K hemagglutination (HA) activity, mediated by the MrkD adhesion located at the tips of type 3 fimbriae. As expected, the Δ*mrkA* mutant failed to mediate a visible MR/K HA reaction at the highest bacterial density tested (approximately 1×10^10^ CFU/mL; Figure 5A). Likewise, the Δ*mrkH* mutant completely lacked MR/K HA activity at the same cell concentration. Upon complementation, strong MR/K HA activity was observed for both strains. The MR/K HA titer for the complemented strains was either reduced to wild-type level (for the Δ*mrkA*-complemented mutant) or to approximately six-fold lower than wild-type level (for the Δ*mrkH*-complemented mutant). It was shown that empty vector controls had no impact on MR/K HA expression for all strains tested (Figure S2). A complementary assay for MR/K HA expression utilized a specific anti-MrkA antibody in immunoblot analysis of total cellular extracts. As expected, MrkA could not be detected in the Δ*mrkA* mutant, while expression was restored when the mutant was complemented with the complete *mrkABCDF* gene cluster (Δ*mrkA* [pMrk]; Figure 5B). Using this assay, MrkA was not detected in wild-type *K. pneumoniae* AJ218, indicating that this strain only weakly expresses type 3 fimbriae in planktonic culture. Similarly, MrkA was not detected in the Δ*mrkH* mutant, but was expressed above wild-type levels when the mutant was complemented with a plasmid expression construct for MrkH (Δ*mrkH* [pMrkH]; Figure 5B). Therefore, when MrkH is over-expressed in *K. pneumoniae*, both the production of the MrkA subunit and the MR/K HA activity is increased. MrkH is therefore a critical, positive regulator of type 3 fimbriae expression.

### Regulation of type 3 fimbriae expression in *K. pneumoniae* by YfiRNB and MrkJ

We also examined the levels of MrkA subunit production and MR/K HA activity of Δ*yfiRNB* (DGC) and Δ*mrkJ* (PDE) mutant strains (Figure 5B). The MrkA subunit was not detected in the Δ*yfiRNB* mutant, but steady-state levels of MrkA were higher than wild-type in mutants complemented with the wild-type *yfiRNB* operon. Conversely, MrkA levels were increased in the Δ*mrkJ* mutant, but absent when complemented with wild-type *mrkJ* gene copies. The MR/K HA activity of these strains was consistent with the immunoblot results (Figure 5A). These observations suggest that YfiRNB and MrkJ function, respectively, as positive and negative regulators of type 3 fimbriae expression. The phenotypes of the mutants are consistent with their roles in modulating the intracellular levels of c-di-GMP.

### MrkI, the LuxR-like regulator, mediates type 3 fimbriae functionality in *K. pneumoniae*

The hemagglutination tests demonstrated that the Δ*mrkI* mutant, shown previously to be deficient in biofilm formation, also had decreased MR/K HA activity. This is consistent with a reduced amount of type 3 fimbriae on the cell surface. These results suggest that MrkI functions as a positive regulator of type 3 fimbriae. Counter-intuitively, the Δ*mrkI* mutant appeared to express more MrkA subunit than the wild-type strain. Moreover, when complemented, the over-expressed MrkI strain appeared to produce even greater amounts of MrkA subunit. Why would the increased levels of MrkA produced in the Δ*mrkI* mutant not be assembled into functional fimbriae to facilitate biofilm formation and MR/K HA?

We hypothesize that this discrepancy is due to MrkI exhibiting multiple roles within the cell. It could function as a minor transcriptional activator of type 3 fimbriae synthesis, in conjunction with a role to regulate another component of the fimbriae assembly pathway (e.g., the usher translocase, MrkB). Alternatively, MrkI could regulate other cell surface factors, such as the polysaccharide capsule, which may in turn affect the function of type 3 fimbriae, as previously described for type 1 fimbriae [19].

In this scenario, a decrease in type 3 fimbriae assembly when MrkI is absent could lead to intracellular accumulation of MrkA, seen as an apparent increase in the steady-state level of MrkA by immunoblot (Figure 5B), resulting in the observed deficiency in MR/K HA and biofilm formation. When MrkI is over-expressed, the functionality of MrkA is restored and type 3 fimbriae expression and biofilm formation is enhanced greater than wild-type levels. The increased levels of MrkA subunit produced in the Δ*mrkI* complemented strain is suggestive of MrkI functioning as a strong activator of *mrkA* transcription when present in high numbers.

**Figure 6:**
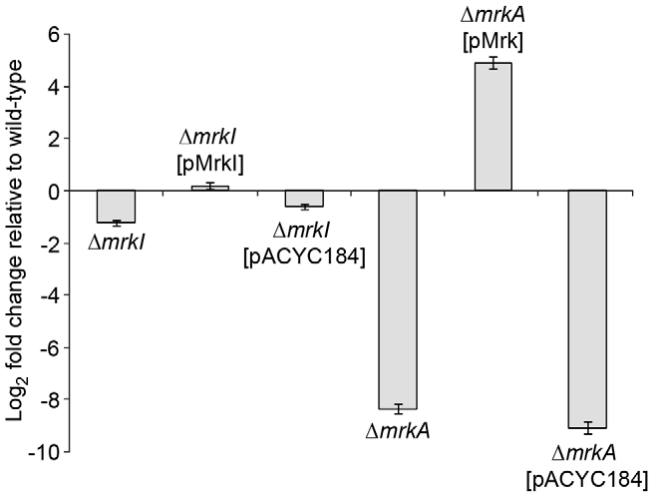
Quantitative RT-PCR analysis of *mrkA* RNA levels. Fold differences in *mrkA* transcript expression levels compared to *K. pneumoniae* AJ218 wild-type levels are shown for the indicated *K. pneumoniae* strains. The *mrkA* transcription expression was normalized to *rpoD* concentrations. Values represent the mean of reactions performed in triplicate. The error bars represent the standard deviation.

Using real-time PCR, only a slight decrease in *mrkA* gene transcript expression is observed in the Δ*mrkI* mutant, consistent with MrkI having a partial role in type 3 fimbriae activation (Figure 6). However, we failed to see a dramatic increase in *mrkA* RNA levels in the Δ*mrkI* complemented strain that could account for the large elevation in MrkA protein production seen in the cell extracts. That MrkB, the periplasmic chaperone, and MrkC, the usher translocase, are required to assemble and export the MrkA protein might account for this. However, we cannot rule out that MrkI also positively regulates other gene(s) that encode factors which participate in the correct folding of the MrkA fimbrial subunit.

### MrkH positively controls the transcription of the *mrkA* promoter

**Figure 7:**
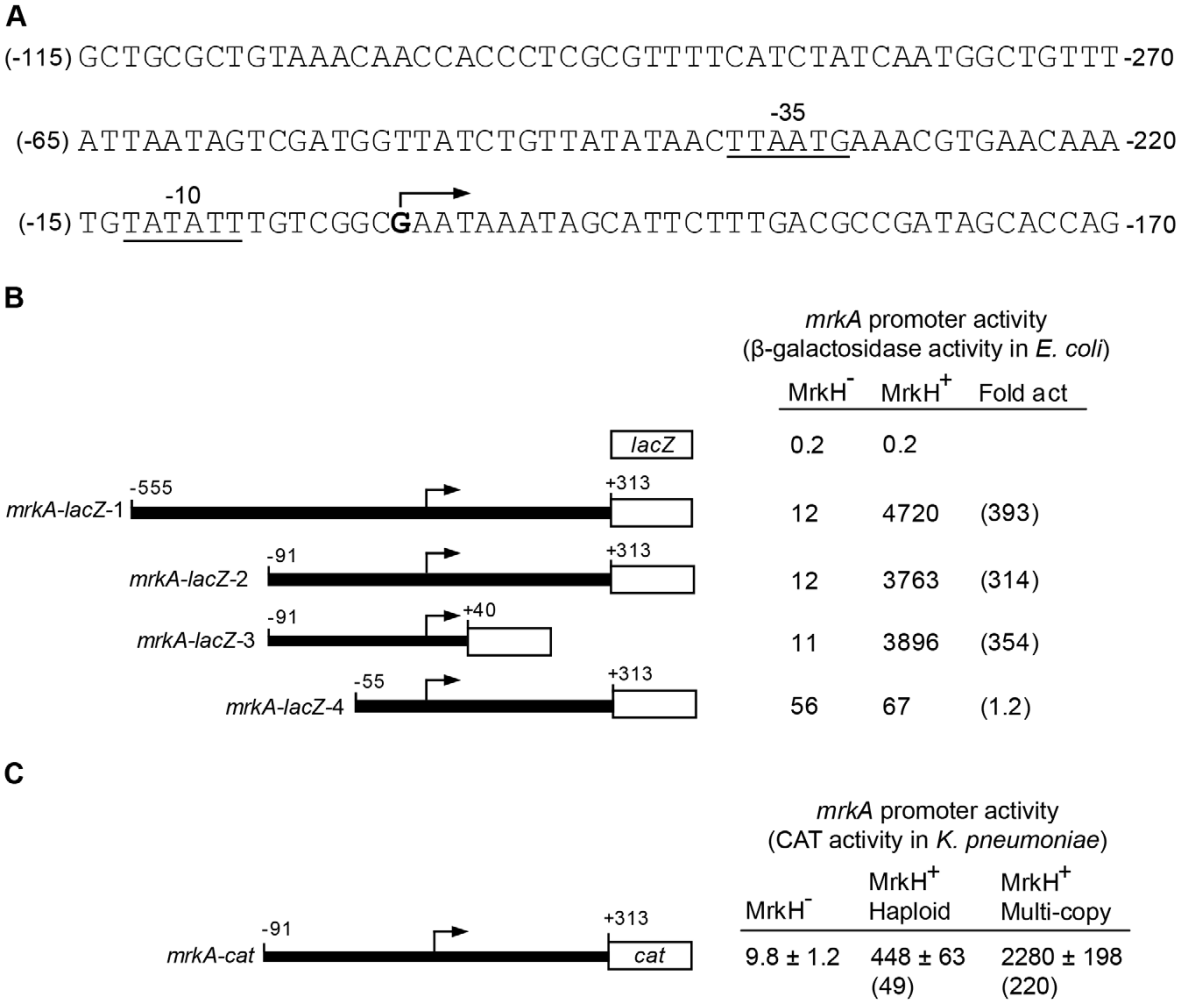
Transcriptional analysis of the *mrkA* regulatory region. (**A**) The nucleotide sequence of the *mrkA* regulatory region is shown. The numbering on the left of the sequence (in brackets) is relative to the transcriptional start site of *mrkA*. The numbering on the right of the sequence is relative to the start codon of the *mrkA* coding sequence. The transcriptional start site is marked with an angled arrow and the putative -35 and -10 regions of the *mrkA* promoter are indicated and underlined. (**B**) Promoter activities of various *mrkA-lacZ* transcriptional fusions in the MrkH^–^ (MC4100 containing pACYC184) and MrkH^+^ (MC4100 containing pMrkH) *E. coli* backgrounds are shown as specific activities of β-galactosidase (Miller) units which are the mean values from three independent assays, with variation <15%. Fold activation (Fold act.) is the specific activity of β-galactosidase of the MrkH^+^ strain divided by that of the MrkH^−^ strain. The numbers shown above the various *mrkA* fragments are relative to the start site of transcription (angled arrows) and the lengths of the various *mrkA* fragments are not to scale. (**C**) The effect of MrkH on transcription of the *mrkA* promoter was analyzed by a CAT assay in three isogenic *K. pneumoniae* backgrounds: MrkH^−^ (Δ*mrkH* + pACYC184-Km^R^), MrkH^+^ haploid (wild-type + pACYC184-Km^R^), and multi-copy MrkH^+^ (Δ*mrkH* + pACYC184-Km^R^-*mrkH*). Specific CAT activities are the averages of three independent assays and the standard deviation values are shown. Values in brackets are fold activation (for details, see above).

We sought to test whether MrkH activates transcription of the *mrkABCDF* operon by stimulating a promoter(s) located in the upstream region of this gene cluster. The sequence of this upstream region is shown in Figure 7A. To test this hypothesis, we established an assay system in *Escherichia coli* using a *lacZ* reporter positioned downstream of specific regulatory regions of the *mrkA* gene (Materials and Methods). Initially, two *mrkA-lacZ* fusions were constructed: *mrkA*-*lacZ*-1 and *mrkA*-*lacZ*-2, spanning positions −759 to +109 (*mrkA-lacZ-*1) and from −295 to +109 (*mrkA-lacZ*-2) relative to the *mrkA* translational start codon. These two plasmids, along with the “promoterless” control plasmid pMU2385, were each transformed into *E. coli* K12 strain MC4100 (Δ*lacZ*) containing either vector pACYC184 (MrkH^–^) or its derivative pMrkH (MrkH^+^) and assayed for β-galactosidase expression.

Compared to the negative control (pMU2385), both *mrkA-lacZ-*1 and *mrkA-lacZ*-2 produced low but significant levels of β-galactosidase activity (12 U) in the MrkH^–^ background (Figure 7B). In both cases, MrkH stimulated expression of *mrkA-lacZ-*1 and *mrkA-lacZ*-2 more than 300-fold. These results demonstrate the presence of a promoter(s) in the upstream region of *mrkA*, which is highly activated by MrkH. Given the similar regulatory patterns of the two constructs, both the promoter and operator elements are present within the 404 bp fragment carried on *mrkA-lacZ*-2.

To verify MrkH-mediated activation of *mrkA* transcription directly in *K. pneumoniae*, the 404 bp *mrkA* fragment (as carried by *mrkA-lacZ*-2) was inserted upstream of the *cat* reporter gene in plasmid pKK232-8. The resulting plasmid, *mrkA-cat*, was then introduced into three isogenic *K. pneumoniae* strains: the wild-type AJ218 strain (MrkH^+^ haploid), the Δ*mrkH* mutant (MrkH^–^) and wild-type AJ218 carrying pMrkH (multi-copy MrkH^+^). As expected, Δ*mrkH* mutants express barely-detectable CAT activity (9.8 U; Figure 7C). In contrast, when *K. pneumoniae* carried the wild-type *mrkH* gene, CAT expression increased 49-fold to 448 U. Further evidence of the stimulatory role of MrkH on transcriptional activation comes from the CAT expression measured (2000 U) when multiple copies of the *mrkH* gene are present.

### Mapping the *mrkA* transcriptional start site and the region required for MrkH activation

**Figure 8:**
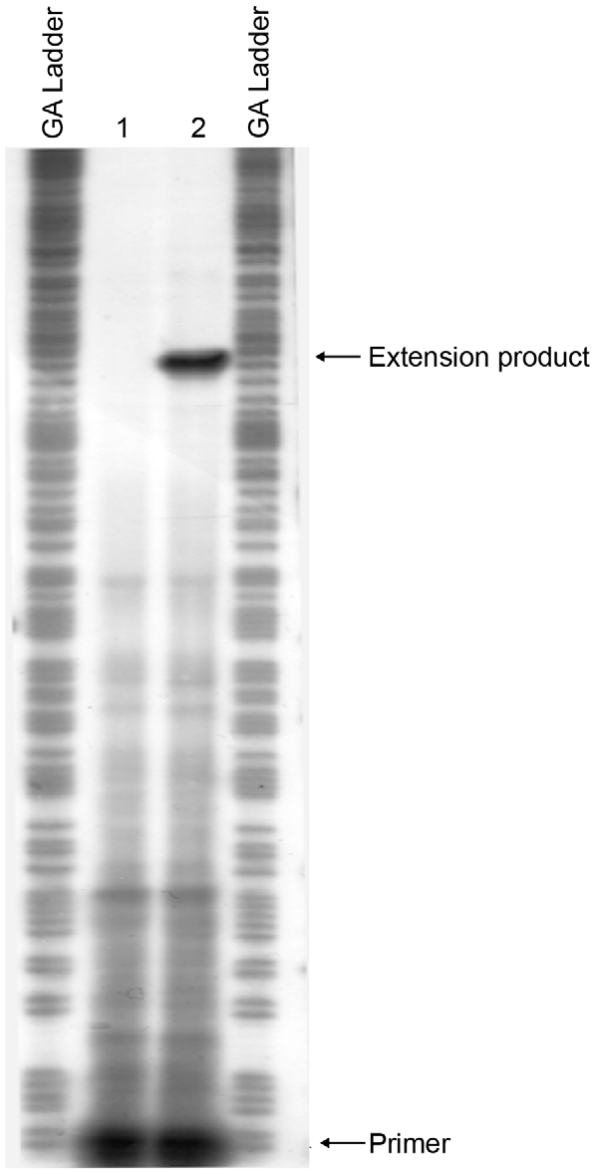
Mapping the start site of transcription of the *mrkA* promoter by primer extension. Total cellular RNA was purified from *E. coli* MC4100 strains containing pMrkH with either pMU2385 (control) or *mrkA-lacZ*-2. The RNA samples were then hybridized with ^32^P-labelled primer Px1mrkARev. Primer extension was performed using AMV reverse transcriptase in the presence of dNTPs. GA Ladder: GA sequence ladder prepared using the *mrkA* PCR fragment generated using primer pairs ^32^P-Px1mrkARev and mrk295F. Lane 1: control experiment using RNA from *E. coli* MC4100 strain containing pMrkH and pMU2385. Lane 2: experiment using RNA from *E. coli* MC4100 strain containing pMrkH and *mrkA-lacZ*-2. The positions corresponding to ^32^P-Px1mrkARev primer and the extension product are marked.

To map the transcriptional start site(s) of *mrkA*, we performed a primer extension experiment. Total cellular RNA was isolated from *E. coli* MC4100 strains containing pMrkH with either pMU2385 (control) or *mrkA-lacZ*-2. Following hybridization of the RNA with ^32^P-labelled primer (Px1mrkARev) and extension with CMV reverse transcriptase in the presence of dNTPs, the samples were analyzed on a sequencing gel. A single extension product was evident from the *mrkA-lacZ*-2 sample (Figure 8). The data mapped the start site of transcription to 204 bp upstream of the putative start site of translation of *mrkA* (Figure 7A). Inspection of the sequence revealed the presence of the hexanucleotides TATATT centered at -10.5 relative to the start site of transcription, which is a good match to the consensus sequence of the -10 region of a bacterial σ^70^ promoter. The only possible -35 sequence (TTAATG), which matches poorly to the consensus sequence, was found 15 bp upstream of the putative -10 region. The combination of a poor -35 region and imperfect spacing may contribute to the very weak promoter activity observed in the MrkH^−^ background.

Mapping the *mrkA* promoter allowed us to make more precise deletion constructs: *mrkA-lacZ*-3 and *mrkA-lacZ*-4, in order to localize the region responsible for MrkH-mediated activation of *mrkA* transcription. As shown in Figure 7B, construct *mrkA-lacZ*-3, in which most nucleotides downstream of the start site of transcription were deleted, exhibited the same degree of activation by MrkH as *mrkA-lacZ*-2, indicating that the deleted downstream sequence (between +40 and +313) was not required for MrkH activation. In the case of *mrkA-lacZ*-4, however, removal of the upstream region between -91 and -56 caused a 5-fold increase in basal level promoter activity in the MrkH^−^ background and completely abolished the MrkH-mediated transcriptional activation of the *mrkA* promoter in the MrkH^+^ background (Figure 7B). From these results, we determined that a *cis*-acting element responsible for MrkH-mediated activation was located between positions -91 and -56.

### C-di-GMP facilitates binding of MrkH to the *mrkA* regulatory region

**Figure 9:**
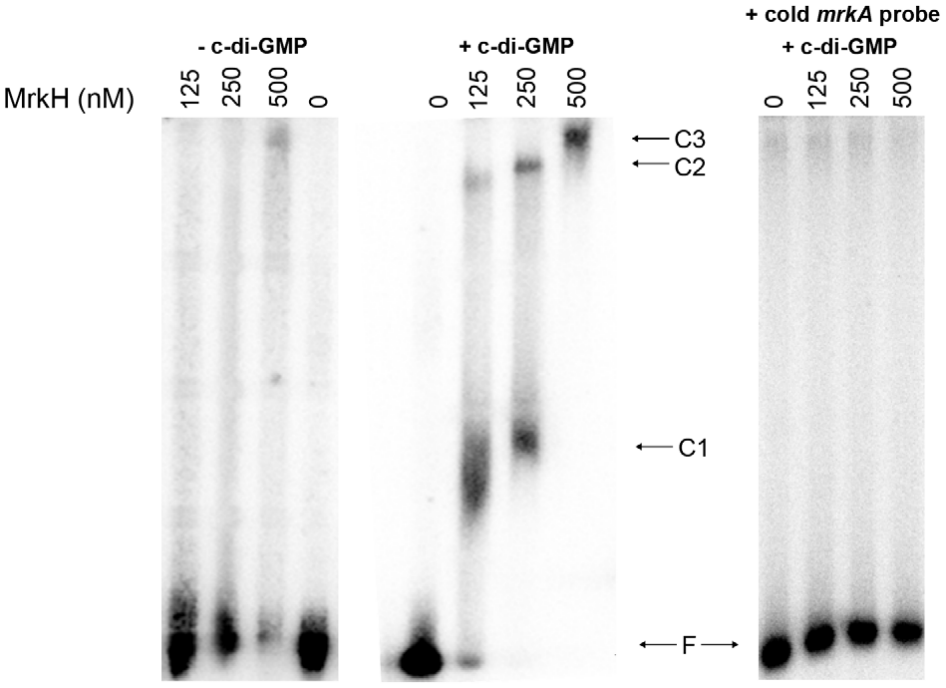
Analysis of the binding of MrkH-8×His to the *mrkA* regulatory region by EMSA. The ^32^P-labelled PCR fragment containing the *mrkA* regulatory region was generated using primer pairs ^32^P-Px1mrkARev and mrk295F. The *mrkA* fragment was mixed with varying amounts of the purified MrkH-8×His protein (from 0 to 500 nM) in the absence or presence of c-di-GMP (200 µM). Following incubation at 30°C for 20 min, the samples were analyzed on native polyacrylamide gels. The right-hand panel shows control reactions with approximately 100-fold molar excess of the unlabeled (cold) *mrkA* promoter fragment (specific competitor DNA), used to demonstrate the specificity of the c-di-GMP-mediated MrkH binding to the *mrkA* promoter region. The unbound DNA (F) and protein-DNA complexes (C1, C2 and C3) are marked.

To test whether MrkH was able to bind directly to the *mrkA* regulatory region, we expressed and purified recombinant MrkH (MrkH-8×His) and used it in an electrophoretic mobility gel shift assay (EMSA). The purity of the MrkH-8×His preparation is shown in Figure S3. The *mrkA* fragment which spanned between −91 and +160, relative to the start site of transcription, was end-labeled with ^32^P and incubated with varying amounts of MrkH-8×His in the absence or presence of 200 µM c-di-GMP at 37°C for 20 min. The samples were then analyzed on native polyacrylamide gels. In the absence of c-di-GMP, no shift of DNA was seen at the MrkH-8×His concentration of 125 nM (Figure 9). Increasing the protein concentration to 250 or 500 nM resulted in the partial shift of DNA; however, no discrete protein-DNA band was obvious. In the presence of c-di-GMP, the majority of the DNA was shifted to form a major protein-DNA complex (C1) and a minor complex (C2) at the MrkH-8×His concentration of 125 nM. At the higher protein concentration of 250 nM, the intensity of the larger C2 complex was enhanced. Increasing the protein concentration to 500 nM led to a complete shift of DNA and the formation of a single, even larger protein-DNA complex (C3; Figure 9). The right panel of Figure 9 showed that the addition of specific cold competitor DNA can out-compete the binding of MrkH to the labeled probe, demonstrating that MrkH binds specifically to the *mrkA* promoter region. Two additional control experiments were carried out in which the *mrkA* promoter fragment was incubated with either the wild-type MrkH-8×His in the presence of GTP or the mutant MrkH-8×His (113R-A, for details see below) in the presence of c-di-GMP. EMSA analysis showed that under these conditions neither the wild-type nor the mutant MrkH can form a protein-DNA complex with the *mrkA* promoter fragment (Figure S4).

The results of EMSA demonstrated that (i) MrkH is a DNA binding protein, (ii) c-di-GMP facilitates the binding of MrkH to the *mrkA* regulatory region and (iii) MrkH can oligomerise on DNA to form a very large MrkH:DNA complex.

### C-di-GMP positively controls the activity of the MrkH protein

**Figure 10:**
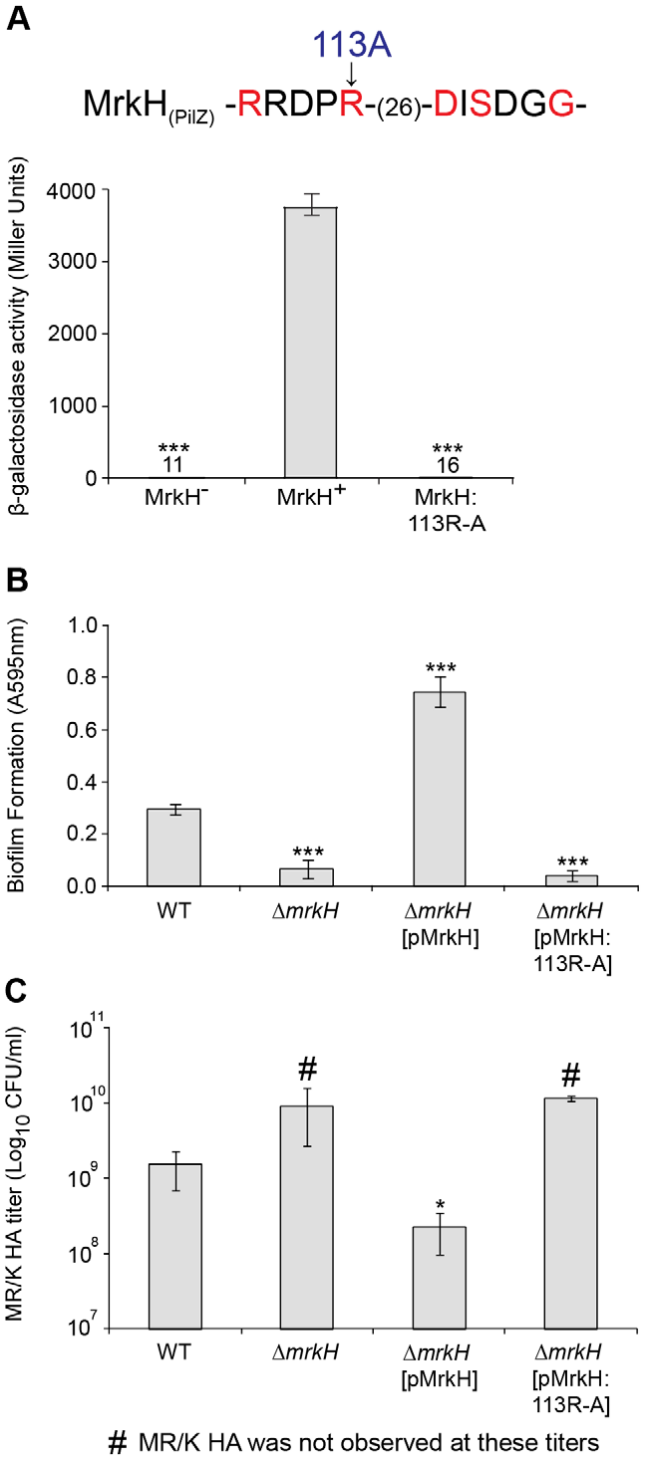
Analysis of a PilZ-domain mutation on MrkH-mediated transcriptional activation, biofilm formation and type 3 fimbriae expression. (**A**) Amino acids within the conserved PilZ domain are shown and residues in red are those known to be critical for c-di-GMP binding in other studies [50]. As indicated, the MrkH mutation carries an arginine to alanine change at position 113. β-galactosidase assays were performed with *E. coli* MC4100 strains: MrkH^−^ (carrying reporter plasmid *mrkA*-*lacZ*-2 and pACYC184), MrkH^+^ (carrying *mrkA*-*lacZ*-2 and pMrkH) and MrkH:113R-A (carrying *mrkA*-*lacZ*-2 and pMrkH:113R-A). The specific β-galactosidase activities are as follows: MrkH^−^: 11±0.9; MrkH^+^: 3760±186 and MrkH:113R-A: 16±1.5. Values represent the mean of three replicate samples. (**B**) Static biofilm formation assay by the indicated *K. pneumoniae* strains. Values represent the mean of four replicate sample wells for each strain performed in two independent experiments. (**C**) MR/K HA assay of the indicated *K. pneumoniae* strains. Values represent the mean of three independent experiments. The error bars represent the standard deviation. Statistical significance between MrkH^+^ and other strains (β-galactosidase assay) was analyzed by the Van der Waerden test, and significance between AJ218 wild-type and Δ*mrkH* strains (biofilm and MR/K HA assays) was analyzed by one-way ANOVA. Tukey HSD post-hoc comparisons are reported, where ***  =  *P*<0.001, *  =  *P*<0.05.

To provide further evidence that c-di-GMP positively regulates MrkH function, we performed mutational analysis of the *mrkH* gene. Construct *mrkH*:113R-A contained a point mutation in which the conserved arginine residue at position 113 within the putative c-di-GMP-binding PilZ domain was replaced with an alanine residue (Figures 4A and 10A). An immunoblot of a C-terminal His_8_-tag fusion with MrkH:113R-A demonstrated that this mutant construct was stably expressed in *E. coli* MC4100 (Figure S5).

To examine the ability of MrkH:113R-A to activate the transcription of *mrkA*, pACYC184 carrying the *mrkH*:113R-A mutation, along with pACYC184 carrying wild-type *mrkH* (MrkH^+^) and pACYC184 alone (MrkH^−^), were transformed into *E. coli* MC4100 containing the single-copy reporter plasmid *mrkA*-*lacZ*-2. These strains were then assayed for β-galactosidase activity following mid-log growth in LB media. As expected, the wild-type MrkH^+^ exerted more than 300-fold activation of the *mrkA* promoter (Figure 10A). Consistent with our prediction that MrkH is essential for c-di-GMP-mediated positive control of *mrkA* transcription, the substitution mutation within the PilZ domain completely destroyed the ability of MrkH to activate transcription from the *mrkA* promoter.

To examine the effect of the PilZ-domain mutation on biofilm formation and type 3 fimbriae expression of *K. pneumoniae* AJ218, we introduced the *mrkH*:113R-A construct described above into the Δ*mrkH* mutant and assayed the resulting strain in static biofilm formation and MR/K HA activity. Although strong biofilm formation (Figure 10B) and MR/K HA activity (Figure 10C) was observed by the Δ*mrkH* mutant upon complementation with wild-type *mrkH*, the *mrkH*:113R-A construct was completely ineffective and failed to rescue the Δ*mrkH* mutant phenotypes.

These results strongly indicated that the c-di-GMP binding region within the PilZ domain of MrkH is critical for transcriptional activation of the *mrk* operon and subsequent biofilm formation via type 3 fimbriae expression.

### MrkH activity is influenced by MrkJ- and YfiN-mediated control of c-di-GMP expression levels

If the above conclusion is correct, we would expect that a decrease in intracellular c-di-GMP levels by enhanced expression of a phosphodiesterase causes a reduction in MrkH activity. Conversely, increasing the endogenous c-di-GMP concentration by enhancing the expression of a diguanylate cyclase would lead to greater MrkH activity.

**Figure 11:**
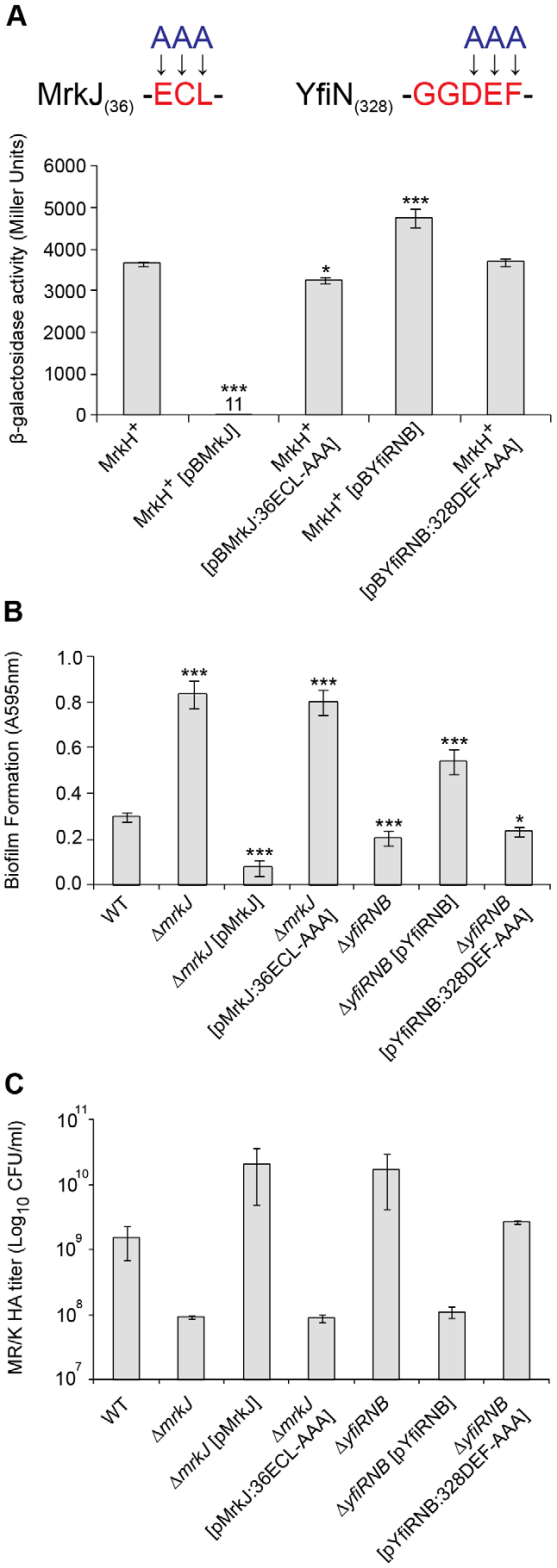
Analysis of EAL- and GGDEF-domain mutations on MrkH-mediated transcriptional activation, biofilm formation and type 3 fimbriae expression. (**A**) Amino acids within the conserved EAL and GGDEF domains of MrkJ and YfiN were substituted with alanine residues. β-galactosidase assays were performed with: *E. coli* MC4100 strain MrkH^+^ (carrying reporter plasmid *mrkA*-*lacZ*-2 and pMrkH), including pBR322-derived vectors pBMrkJ (wild-type), pBMrkJ:36ECL-AAA, pBYfiRNB (wild-type) or pBYfiRNB:328DEF-AAA. Values represent the mean of three replicate samples. (**B**) Static biofilm formation assay by the indicated *K. pneumoniae* strains. Values represent the mean of four replicate sample wells for each strain performed in two independent experiments. (**C**) MR/K HA assay of the indicated *K. pneumoniae* strains. Values represent the mean of three independent experiments. The error bars represent the standard deviation. Statistical significance between MrkH^+^ (wild-type) and other strains (β-galactosidase assay), as well as AJ218 wild-type and isogenic mutant strains (MR/K HA assay) was analyzed by the Van der Waerden test. Significance between AJ218 wild-type and isogenic mutant strains (biofilm assay) was analyzed by one-way ANOVA. Tukey HSD post-hoc comparisons are reported, where ***  =  *P*<0.001, *  =  *P*<0.05.

To test this hypothesis, we introduced pBR322 derivatives carrying either the wild-type *mrkJ* gene, the *yfiRNB* operon, or pBR322 alone into *E. coli* MC4100, which contained pACYC184 carrying wild-type *mrkH* (MrkH^+^) and the reporter plasmid *mrkA*-*lacZ*-2. To confirm the roles of MrkJ and YfiN as c-di-GMP-specific phosphodiesterase and diguanylate cyclase, respectively, we also generated site-directed mutant constructs of these enzymes in their conserved c-di-GMP hydrolysis/synthesis catalytic sites and tested their activity alongside the wild-type constructs. Construct *mrkJ:*36ECL-AAA contained substitution mutations in which the EAL domain of MrkJ (starting at residue 36) was replaced with alanine residues (Figures 4B and 11A). In addition, construct *yfiRNB*:328DEF-AAA carried substitution mutations in which the DEF residues of the GGDEF domain of YfiN (starting at residue 328) were replaced with alanine residues (Figures 4C and 11A).

These strains were then assayed for β-galactosidase activity following mid-log growth in LB media. Enhanced expression of wild-type MrkJ led to a complete inability of MrkH to activate transcription from the *mrkA* promoter (Figure 11A). Conversely, mutation of the EAL domain of MrkJ led to enhanced MrkH-mediated transcriptional activation. This result suggested that an inactive EAL domain renders MrkJ unable to effectively hydrolyze c-di-GMP, permitting c-di-GMP accumulation and increased MrkH activity. The increased expression of wild-type YfiRNB resulted in a small but significant enhancement in MrkH-mediated activation of *mrkA* transcription. However, this increase was not observed from the YfiN construct containing the mutated GGDEF domain, suggesting that the lowered MrkH-mediated activation of the *mrkA* promoter was caused by YfiN impairment in c-di-GMP synthesis. The site-directed mutations of the EAL and GGDEF domains of MrkJ and YfiN, respectively, also had pronounced effects on biofilm formation and type 3 fimbriae synthesis by *K. pneumoniae* AJ218. The *mrkJ:*36ECL-AAA and *yfiRNB*:328DEF-AAA gene constructs also failed to effectively complement the respective Δ*mrkJ* and Δ*yfiRNB* mutant strains in static biofilm formation (Figure 11B) and MR/K HA activity (Figure 11C).

Taken together, these results indicate that MrkJ and YfiN are c-di-GMP-specific phosphodiesterase and diguanylate cyclase, respectively. Moreover, we have clearly demonstrated that c-di-GMP is a positive cofactor essential for MrkH-mediated transcriptional activation of the *mrk* operon for type 3 fimbriae synthesis and biofilm formation by *K. pneumoniae*.

### Detection of phosphodiesterase activity for the MrkJ protein

**Figure 12:**
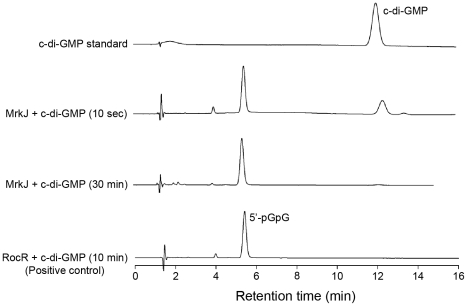
MrkJ displays strong phosphodiesterase activity. C-di-GMP hydrolysis by purified MrkJ was analyzed by HPLC. The formation of 5′-pGpG was monitored at various time-points and the c-di-GMP-specific phosphodiesterase RocR [94] was used as the positive control.

From the data presented, MrkJ represents a c-di-GMP-specific phosphodiesterase that has pronounced negative effects on *K. pneumoniae* biofilm formation. To further characterize the kinetics of c-di-GMP hydrolysis by MrkJ, we expressed and purified MrkJ (MrkJ-8×His) and analyzed its enzyme activity by High-Performance Liquid Chromatography (HPLC). The purity of the MrkJ-8×His preparation is shown in Figure S6. We demonstrated that a significant amount of c-di-GMP was hydrolyzed by MrkJ-8×His to form the degradation product 5′-pGpG after 10 sec (Figure 12). The c-di-GMP was completely converted to 5′-pGpG after 30 min. These results confirmed that MrkJ possesses very strong phosphodiesterase activity, a significant feature for an inhibitor of biofilm formation.

## Discussion

### The role of type 3 fimbriae in *K. pneumoniae*

The type 3 fimbriae of *K. pneumoniae*, encoded by the *mrkABCDF* operon, represent a surface appendage that is significant in mediating biofilm formation. As the major pilin, MrkA is required to extend the fimbriae [8], [9], which is particularly important in the case of *K. pneumoniae* given the thick capsule surrounding the outer membrane [19]. Past studies have shown that Δ*mrkA* mutants have a greatly diminished capacity for adherence to abiotic surfaces and for biofilm formation in continuous flow-through chambers [14], [15], [26]. We found that during planktonic growth very little MrkA is produced, with only a sensitive functional assay able to detect fimbria-dependent hemagglutination. However, the type 3 fimbriae are essential for biofilm growth of *K. pneumoniae* AJ218 in flow-cell chambers and adherence to PVC surfaces, a common material used in indwelling medical devices, such as catheters and endotracheal tubes.

Type 3 fimbriae are found in several groups of *Enterobacteriaceae* [27], and are extended by a step-wise addition of MrkA subunits to the base of the filament [13], [28]. Each MrkA subunit is taken up by the N-terminal domain of the Usher translocase, with the translocase thereby catalyzing polymerization and providing the pore through which the filament passes across the outer membrane. In *K. pneumoniae,* these fimbriae must extend from the outer membrane through a thick carbohydrate capsule to reach, and extend beyond, the actual cell surface. Comparative genomics and experimental evidence suggests that most clinical and environmental strains of *K. pneumoniae* carry the genes required to produce type 3 fimbriae [29], [30]. The *K. pneumoniae* AJ218 *mrkABCDF* operon is almost identical in nucleotide sequence to other *K. pneumoniae* clinical strains. This suggests that differences in the biofilm-forming abilities between type 3 fimbriae-expressing isolates is a consequence of variation in fimbriae regulation/expression mechanisms and/or the involvement of other biofilm factors, such as the capsule polysaccharide.

### Intracellular levels of c-di-GMP coordinate type 3 fimbriae extension in *K. pneumoniae*

The best-studied usher-assembled pili/fimbriae are represented by the P (*pap*) pili and type 1 (*fim*) fimbriae, both of which are controlled by phase variation mechanisms ensuring their expression is either switched “ON” or “OFF” [13]. In the *pap* system, local and global DNA binding regulators direct phase switching through modulating DNA methylation patterns [31]. Phase variable expression of type 1 fimbriae in *E. coli* is coordinated by the inversion of a *fimS* DNA element located upstream of the *fim* operon, via site-specific recombinase-like integrases (FimB and FimE) [32], [33].

Quite unlike previously studied systems, the type 3 fimbriae of *K. pneumoniae* are regulated by c-di-GMP. A recent study identified MrkJ as a phosphodiesterase and demonstrated the activity of the purified protein in hydrolyzing c-di-GMP [34], and we demonstrated by HPLC analysis that MrkJ exhibits very strong phosphodiesterase activity. C-di-GMP was first identified as an allosteric regulator of cellulose synthase in *Gluconacetobacter xylinus* [35], [36], [37]. Since then, c-di-GMP has been characterized as an intracellular second messenger, ubiquitous in prokaryotes [38], [39], [40]. This important signaling molecule regulates a variety of processes, including motility, extracellular polysaccharide synthesis, cell differentiation and virulence gene expression [41], [42]. Deletion of *mrkJ* resulted in an increase in type 3 fimbriae production and biofilm formation as a result of the accumulation of intracellular c-di-GMP [34]. Furthermore, our transposon library screen has provided a mechanistic basis for understanding how c-di-GMP signaling controls biofilm formation and type 3 fimbriae in this emergent pathogen, given the opposing phenotypes of mutants defective in the GGDEF domain-containing diguanylate cyclase (YfiN) and EAL domain-containing phosphodiesterase (MrkJ), and the role of c-di-GMP in regulating the transcriptional activation mediated by the novel DNA-binding protein MrkH. Apart from MrkH, all three sequenced *K. pneumoniae* genomes contain only one other PilZ-domain protein – BcsA, the catalytic subunit of cellulose synthase. In addition, like most bacteria, *K. pneumoniae* harbours multiple GGDEF and EAL proteins (12 GGDEF, 9 EAL and 6 GGDEF-EAL). Rather than implicated in redundancy, these protein domains are usually coupled to sensory input or information transfer domains, and are thus believed to carry out specific tasks in the cell.

Some of the elements we have identified in the c-di-GMP-mediated ‘biofilm switch’ in *K. pneumoniae* have been reported in other bacteria, suggesting that at a cellular level, such a switch may represent a general control mechanism. For example, the *Pseudomonas aeruginosa* Cup (*cup*) fimbriae, assembled through the chaperone-usher pathway, are down-regulated at the transcriptional level indirectly by phosphodiesterase response regulators [43]. Furthermore, in *E. coli*, type 1 fimbriae expression in the adherent-invasive strain LF82 is also regulated, at least in part, via a c-di-GMP-dependent pathway [44].

The environmental signals that regulate YfiRNB expression to modulate type 3 fimbriae expression and hence biofilm formation, are unknown. In *P. aeruginosa*, it has been suggested that YfiB, which belongs to the Pal (peptidoglycan-associated lipoprotein) family of lipoproteins, could relay stress response signals from the outer envelope to YfiN via YfiR [45]. It is also understood that the periplasmic tyrosine phosphatase (TpbA), which responds to quorum sensing signals, can dephosphorylate a tyrosine residue located within the periplasmic domain of YfiN (termed TpbB) leading to deactivation of the GGDEF domain and c-di-GMP repression [46]. Whether analogous systems exist for the YfiRNB homolog in *K. pneumoniae* to control c-di-GMP levels, biofilm formation and type 3 fimbriae expression remains to be determined.

MrkH is predicted to contain a PilZ domain, widely distributed in bacterial proteins and responsible for c-di-GMP binding, which controls a range of c-di-GMP-mediated cellular functions [38], [47]. Several lines of evidence suggest that MrkH is a c-di-GMP effector protein. Firstly, the C-terminal PilZ domain of MrkH has all of the conserved residues known to be crucial for c-di-GMP binding. Secondly, changing the conversed arginine residue at position 113 within the putative c-di-GMP-binding domain of MrkH completely destroyed the ability of MrkH to activate the transcription from the *mrkA* promoter *in vivo*. Thirdly, the role of MrkH is in agreement with the phenotypes observed from mutational and over-expression experiments conducted with the c-di-GMP turnover enzymes YfiN and MrkJ. Fourthly, DNA-binding by purified MrkH is strongly stimulated in the presence of c-di-GMP.

### MrkH is a novel transcriptional activator of the *mrk* gene cluster in *K. pneumoniae*

Transcriptional analysis using promoter-reporter fusions and a primer extension assay identified a single σ^70^ promoter (the *mrkA* promoter) preceding the *mrkABCDF* operon. Although the putative -10 region (TATATT) of this promoter matches well with the consensus sequence, the putative -35 region (TTAATG) and the spacer between the two regions (15 bp) are poorly conserved. The -35 region of σ^70^ promoters is recognized and contacted by region 4 of the RNA polymerase σ^70^ subunit during the first step of transcription initiation to form a closed complex [48]. With respect to the *mrkA* promoter, the presence of the poorly conserved -35 region and the shorter spacer (15 bp), which causes the imperfect alignment of the -35 and -10 regions, is the most likely explanation for the weak interaction of RNA polymerase with the promoter DNA and the resultant low basal levels of promoter activity that were observed in a MrkH^–^ background. Remarkably, this weak promoter can be induced up to several hundred fold in bacterial strains expressing the MrkH protein, through the binding of the activator to a *cis*-acting element immediately upstream of the -35 region. The MrkH binding-site resembles the Class 1 Crp-dependent promoters of *E. coli*, where Crp activates transcription initiation by recruiting RNA polymerase to the promoter via a direct contact between Crp and the σ subunit of RNA polymerase [49].

### MrkH is activated in the presence of c-di-GMP to ‘switch on’ biofilm formation

The PilZ domain characteristically undergoes significant structural changes upon c-di-GMP binding, allosterically activating the protein to interact with downstream effectors [50], [51], [52], [53]. We suggest that c-di-GMP binding to the PilZ domain of MrkH causes structural changes to the protein and activation of an output domain, specifically to permit DNA-binding of MrkH immediately upstream of the -35 box of the *mrkA* promoter. This complex could then stabilize or recruit RNA polymerase to the promoter for transcriptional initiation of the *mrkABCDF* operon, thereby effectively ‘switching on’ type 3 fimbriae production and biofilm formation. MrkH could represent a novel type of DNA-binding protein: it has no obvious homology to a HTH DNA-binding domain, which is most commonly used for DNA-binding by bacterial regulatory proteins. However, the N-terminal portion of the MrkH protein (the first 106 aa) is predicted by secondary structure prediction programs (such as PSIPRED [54]) to constitute five β strands flanked by two α helices which is similar to that of a bacterial LytTR DNA-binding domain [55], [56]. LytTR-domain proteins account for approximately 3% of all prokaryotic response regulators [57] and members include the *Staphylococcus aureus* AgrA, a global regulator of virulence [58], [59]; *P. aeruginosa* AlgR, which modulates the production of alginate [60]; and, interestingly, *K. pneumoniae* MrkE, a putative regulatory protein encoded on a plasmid next to the *mrkABCDF* gene cluster from isolate IA565 [9]. It is also suggested that a LytTR domain is structurally homologous to the well-characterized DNA-binding domain of the Sac7d protein from *Sulfolobus acidocaldarius*, which is comprised of five β strands followed by an α helix [55], [61], [62]. Although there is a lack of sequence similarity between various characterized LytTR DNA-binding motifs, the LytTR domain appears to share a common secondary structure which gives rise to a unique fold. We are currently investigating the structural and functional basis of any possible DNA-binding motif of MrkH.

C-di-GMP participates in the transcriptional regulation of other regulatory proteins. The transcriptional regulator VpsT from *V. cholerae* is a master regulator of biofilm formation that binds c-di-GMP (via a conserved W[F/L/M][T/S]R sequence motif) to inversely control extracellular matrix production and motility [63]. In the plant pathogen, *Xanthomonas axonopodis*, c-di-GMP allosterically represses the activity of Clp, a global transcriptional regulator of about 300 genes involved in pathogenesis [64], [65]. C-di-GMP association with the cyclic nucleotide monophosphate (cNMP) binding domain of Clp results in conformational changes that abolish the binding of Clp to its DNA targets. In *P. aeruginosa*, c-di-GMP binding to FleQ, a transcriptional repressor of the *pel* operon involved in exopolysaccharide substance production, inactivates its DNA binding ability to upregulate EPS biosynthesis [66], [67].

Although c-di-GMP has been implicated in numerous studies to mediate biofilm formation, this is the first demonstration that this compound can work as an effector that stimulates the DNA-binding ability of a PilZ domain-containing transcriptional activator. Therefore, our study identified the MrkH regulator as a missing link between the second messenger c-di-GMP and the formation of biofilms in *K. pneumoniae*. The dependence of type 3 fimbrial synthesis on the MrkH transcriptional activator, with its activity directly controlled by c-di-GMP via the expression of diguanylate cyclase and phosphodiesterase enzymes, permits an elegantly and tightly controlled mechanism by which *K. pneumoniae* can quickly regulate the switch between the planktonic and biofilm lifestyles when the relevant environmental sensors are triggered.

Close homologs of the *mrkHIJ*-*ABCDF* cluster are present in the *C. koseri* BAA-895 genome. Examination of the *C. koseri* MrkH, MrkI and MrkJ homologs revealed conservation of the PilZ, LuxR and EAL domains, respectively (data not shown). A close homolog of YfiN carrying a conserved GGDEF domain was also found (data not shown). *C. koseri*, a Gram-negative enterobacterium, is a cause of urinary tract infections, sepsis and meningitis, predominately amongst neonates and immunocompromised adults [68]. The habitat and epidemiology of this pathogen closely parallels that of *K. pneumoniae*, and expression of type 3 fimbriae to facilitate biofilm formation has been demonstrated [24], [69]. Given these similarities between the two pathogens, we speculate that the regulation of type 3 fimbriae expression in *C. koseri* might involve analogous mechanisms to those described here for *K. pneumoniae*.

### MrkI is a candidate regulator of *K. pneumoniae* type 3 fimbriae function and expression

The MrkI regulator belongs to a family of transcriptional regulators (FixJ/LuxR/UhpA) that contain a C-terminal HTH DNA-binding motif and a receiver domain at the N-terminal region [70]. CsgD, the analogous protein found in *Salmonella enterica* serovar Typhimurium and *E. coli*, is a master regulator of the multicellular (rdar) morphotype of *S. typhimurium* [71] and acts by positively regulating two extracellular matrix components: curli fimbriae, via activation of the *csgBAC* operon [72], [73] and cellulose, via activation of the DGC-encoding gene *adrA* [74], [75]. The VspT regulator of *V. cholerae* described above is a member of the FixJ/LuxR/CsgD family of response regulators and consists of an N-terminal receiver domain and C-terminal HTH DNA-binding domain [63]. The c-di-GMP binding motif of VspT was found to be absent in MrkI, however.

We propose that MrkI, which is co-coordinately expressed with MrkH, possesses multiple functions to both regulate the expression and functionality of type 3 fimbriae. As a minor activator of type 3 fimbriae expression, at least three mechanisms are possible, including self-transcriptional activation with *mrkH* to increase c-di-GMP effector protein production, activation of DGC-encoding gene(s) to stimulate c-di-GMP production, or regulation of the fimbriae themselves through partial activation of the *mrkABCDF* operon. Thus, while we have demonstrated that MrkI is necessary for expression of functional type 3 fimbriae, further studies are required to determine which gene(s) are directly regulated by MrkI to eventuate in increased expression of type 3 fimbriae.

### Implications and future research

**Figure 13:**
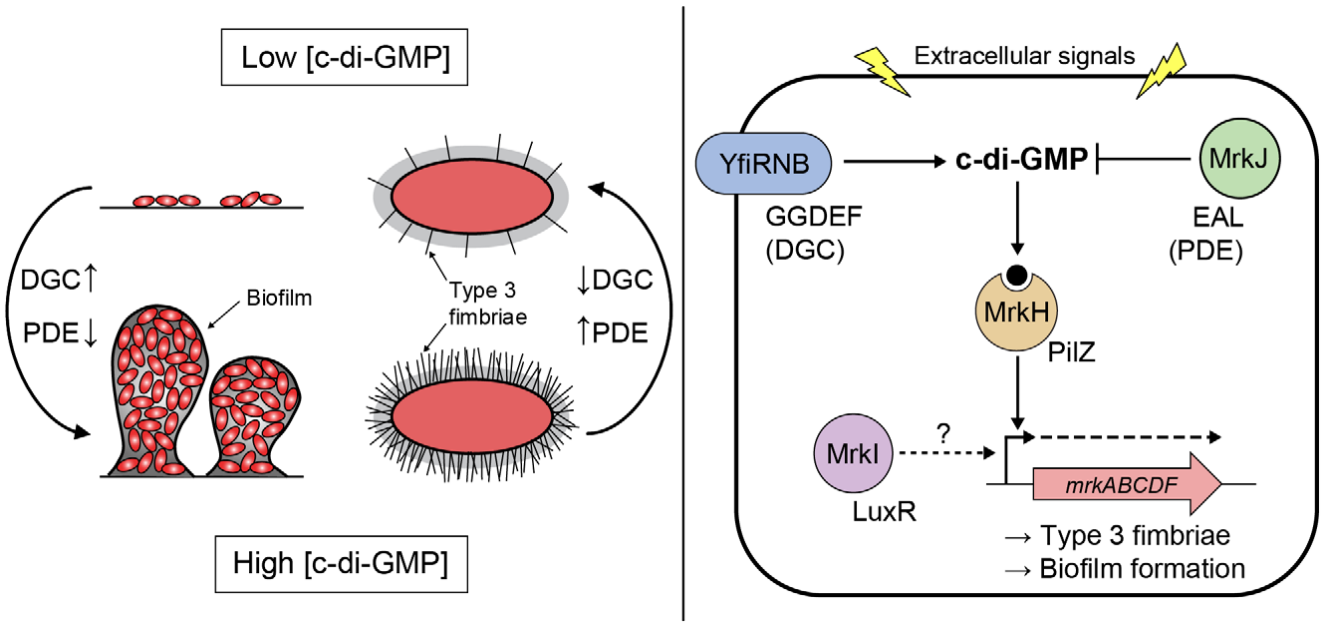
Model of c-di-GMP-mediated control of type 3 fimbriae expression and biofilm formation in *K. pneumoniae*. Signals resulting in increased/decreased intracellular concentration of c-di-GMP, via changes in the relative activities of DGCs (carrying a GGDEF domain [YfiN]) and PDEs (carrying an EAL domain [MrkJ]), direct the DNA-binding activity of the c-di-GMP receptor MrkH (carrying a PilZ domain). High c-di-GMP levels promote biofilm formation through MrkH:c-di-GMP-dependent transcriptional activation of the *mrkABCDF* operon encoding type 3 fimbriae. The major pilin subunit MrkA is bound by the MrkB chaperone in the periplasm to activate it for polymerization by the usher translocase MrkC. Once sufficiently elongated, the fimbriae would emerge through the extracellular capsule layer (represented by grey shading surrounding the cell). Conversely, low c-di-GMP levels promote biofilm dispersal and the planktonic state through a decrease in activated MrkH:c-di-GMP availability. Whether the LuxR-like regulator MrkI acts on *mrkABCDF* gene expression, or the expression of other factors influencing type 3 fimbriae polymerization or capsule polysaccharide production remains to be determined.

The genetic screen reported here revealed the significance of c-di-GMP-mediated regulation of biofilm formation and type 3 fimbriae expression in *K. pneumoniae* (Figure 13). Type 3 fimbriae are important for efficient surface attachment and biofilm formation and are likely to be a primary virulence factor, with biofilms implicated as a significant cause of hospital-acquired infections and related deaths [76], [77]. Indwelling medical devices, such as urinary catheters, provide an attractive surface for bacterial pathogens such as *K. pneumoniae* to establish biofilms for colonization within human hosts. Future research lies not only in understanding how c-di-GMP effector proteins relay upstream signals to control phenotypes involved in biofilm formation, but in identifying the environmental signals that trigger c-di-GMP synthesis and breakdown. Moreover, c-di-GMP regulation and recognition within bacteria offer new targets for strategic intervention, for example the development of novel inhibitors that could be incorporated into the materials used to produce hospital devices.

## Materials and Methods

### Bacterial strains, plasmids and growth conditions

The bacterial strains and plasmids used in this study are described in Table 2. *K. pneumoniae* strain AJ218 (capsule serotype K54) is a human, urinary tract infection isolate [20]. *K. pneumoniae* AJ218^Rif^ is a spontaneous rifampacin resistant strain used in conjugation experiments to create the transposon mutant library. *E. coli* DH5α was used for cloning purposes [78]. Unless otherwise stated, bacteria were maintained in Luria-Bertani (LB) medium overnight at 37°C with shaking. When appropriate, media were supplemented with antibiotics at the following concentrations: ampicillin (Ap), 100 µg/mL; kanamycin (Km), 50 µg/mL; chloramphenicol (Chl), 30 µg/mL (for *E. coli* DH5α) and 80 µg/mL (for *K. pneumoniae*); rifampacin (Rif), 150 µg/mL; trimethoprim (Tm), 40 µg/mL.

**Table 2:** Bacterial strains and plasmids used in this study.

Strain or plasmid	Relevant phenotypes and genotypes	Source or reference
***K. pneumoniae***		
AJ218	Wild-type, clinical isolate, serotype K54; Ap^R^	[20]
AJ218^Rif^	AJ218; Rif^R^ Ap^R^	This study
JW30A8	AJ218^Rif^ mini-Tn*5* mutant *mrkA*::Tn*5Km2*; Ap^R^ Km^R^	This study
JW69A4	AJ218^Rif^ mini-Tn*5* mutant *mrkB*::Tn*5Km2*; Ap^R^ Km^R^	This study
JW8E7	AJ218^Rif^ mini-Tn*5* mutant *mrkC*::Tn*5Km2*; Ap^R^ Km^R^	This study
JW34H5	AJ218^Rif^ mini-Tn*5* mutant *mrkH*::Tn*5Km2*; Ap^R^ Km^R^	This study
JW64B4	AJ218^Rif^ mini-Tn*5* mutant *mrkI*::Tn*5Km2*; Ap^R^ Km^R^	This study
JW45H9	AJ218^Rif^ mini-Tn*5* mutant *mrkJ*::Tn*5Km2*; Ap^R^ Km^R^	This study
JW34D8	AJ218^Rif^ mini-Tn*5* mutant *yfiN*::Tn*5Km2*; Ap^R^ Km^R^	This study
JW100	AJ218 deletion mutant Δ*mrkA*::*km*; Ap^R^ Km^R^	This study
JW133	AJ218 deletion mutant Δ*mrkH*; Ap^R^	This study
JW96	AJ218 deletion mutant Δ*mrkI*::*km*; Ap^R^ Km^R^	This study
JW99	AJ218 deletion mutant Δ*mrkJ*::*km*; Ap^R^ Km^R^	This study
JW98	AJ218 deletion mutant Δ*yfiRNB*::*km*; Ap^R^ Km^R^	This study
***E. coli***		
S17 λ*pir*	*recA thi pro hsdR M* ^+^ RP4::2-Tc::Mu::Km Tn*7* λ*pir* lysogen; Tp^R^ Sm^R^	[95]
DH5α	F- *endA1 hsdR17* (r_k_^−^, m_k_^+^) *supE44 thi-1* λ^−^ *recA1 gyrA96 relA1 deoR* Δ(*lacZYA-argF*)-U169 Φ80d*lac*ZΔM15; Nal^R^	[78]
MC4100	Δ(*argF-lac*)*U169, rpsL150*, *relA*, *araD139, fib5301*, *deoC1, ptsF25*	[96]
BL21(DE3)	F-, *omp*T, *hsd*S_B_(r_B_-, m_B_-), *dcm*, *gal*, λ(DE3)	[97]
**Plasmids**		
pMrk	AJ218 *mrkABCDF* cloned into pACYC184; Chl^R^	This study
pMrkH	AJ218 *mrkH* cloned into pACYC184; Chl^R^	This study
pMrkI	AJ218 *mrkI* cloned into pACYC184; Chl^R^	This study
pMrkJ	AJ218 *mrkJ* cloned into pACYC184; Chl^R^	This study
pYfiRNB	AJ218 *yfiRNB* cloned into pACYC184; Chl^R^	This study
pACYC184	Medium-copy-no. cloning vector, p15A ori; Tet^R^ Chl^R^	[98]
pBR322	Medium-copy-no. cloning vector, pMB1 ori; Tet^R^ Ap^R^	[99]
pGEM-T Easy	High-copy-no. cloning vector for PCR products; Ap^R^	Promega
TOPO-TA	High-copy-no. cloning vector for PCR products; Ap^R^ Km^R^	Invitrogen
pUT-miniTn*5Km2*	Mini-Tn*5Km2* transposon in the pUT vector; Ap^R^ Km^R^	[100]
pKD4	Source of FRT-flanked Kan^R^ cassette; Ap^R^ Km^R^	[80]
pACBSR	Ara promoter control, I-*Sce*I and λ Red recombinase; Chl^R^	[79]
pCP20	Flp helper plasmid, temp-sensitive replication; Ap^R^ Chl^R^	[81]
pMU2385	*galK'-lac'Z*, IncW, single-copy-no. transcriptional-fusion vector; Tp^R^	[101]
pKK232.8	pBR322 derivative used for making promoter-*cat* fusions; Ap^R^ Chl^R^	Amersham
*mrkA-lacZ*-1	*mrkA*-*lacZ* transcriptional fusion vector (pMU2385) from *mrkA* promoter nucleotides −759 to +109 relative to the *mrkA* start codon; Tp^R^	This study
*mrkA-lacZ*-2	*mrkA*-*lacZ* transcriptional fusion vector (pMU2385) from *mrkA* promoter nucleotides −295 to +109 relative to the *mrkA* start codon; Tp^R^	This study
*mrkA-lacZ*-3	*mrkA*-*lacZ* transcriptional fusion vector (pMU2385) from *mrkA* promoter nucleotides −295 to −165 relative to the *mrkA* start codon; Tp^R^	This study
*mrkA-lacZ*-4	*mrkA*-*lacZ* transcriptional fusion vector (pMU2385) from *mrkA* promoter nucleotides −259 to +109 relative to the *mrkA* start codon; Tp^R^	This study
*mrkA-cat*	*mrkA*-*cat* transcriptional fusion vector (pKK232.8) from *mrkA* promoter nucleotides −295 to +109 relative to the *mrkA* start codon; Ap^R^ Chl^R^	This study
pET11a	Expression vector using T7 promoter; Ap^R^	Novagen
pET11a-mrkH-8His	AJ218 *mrkH* tagged with 8×His at C-terminus cloned into pET11a; Ap^R^	This study
pET11a-mrkH(113R-A)-8His	AJ218 *mrkH*:113R-A tagged with 8×His at C-terminus cloned into pET11a; Ap^R^	This study
pET11a-mrkJ-8His	AJ218 *mrkJ* tagged with 8×His at C-terminus cloned into pET11a; Ap^R^	This study
pGMrkH-8His	AJ218 *mrkH* tagged with 8×His at C-terminus cloned into pGEM-T Easy; Ap^R^	This study
pGMrkH(113-R)-8His	AJ218 *mrkH* carrying R→A amino acid substitution at residue position 113 tagged with 8×His at C-terminus cloned into pGEM-T Easy; Ap^R^	This study
pMrkH(113R-A)	AJ218 *mrkH* carrying R→A amino acid substitution at residue position 113 cloned into pACYC184; Chl^R^	This study
pMrkJ(36ECL-AAA)	AJ218 *mrkJ* carrying ECL→AAA amino acid substitution starting at residue position 36 cloned into pACYC184; Chl^R^	This study
pYfiRNB(328DEF-AAA)	AJ218 *yfiRNB* carrying DEF→AAA amino acid substitution starting at residue position 328 of YfiN cloned into pACYC184; Chl^R^	This study
pBMrkJ(36ECL-AAA)	AJ218 *mrkJ* carrying ECL→AAA amino acid substitution starting at residue position 36 cloned into pBR322; Ap^R^	This study
pBYfiRNB(328DEF-AAA)	AJ218 *yfiRNB* carrying DEF→AAA amino acid substitution starting at residue position 328 of YfiN cloned into pBR322; Ap^R^	This study

### DNA manipulation techniques

PCR amplifications were performed using GoTaq Green Master Mix (Promega, Madison, WI), Phusion Flash High-Fidelity PCR Master Mix (Finnzymes, Finland) or Vent DNA Polymerase (New England Biolabs, Ipswich, MA). Restriction endonucleases and T4 DNA ligase were obtained from New England Biolabs. Synthetic oligonucleotides for PCR and sequencing (Table S1) were obtained from GeneWorks (Hindmarsh, South Australia, Australia).

### Transposon mutagenesis and Y-linker ligation PCR

Mid-log cultures of donor (*E. coli* S17-λ*pir* harboring suicide vector pUT-mini-Tn*5Km2*) and recipient (*K. pneumoniae* AJ218^Rif^) were mixed in a 1∶1 ratio in a final volume of 1 mL. The conjugation mix was then centrifuged, resuspended in 0.1 mL LB and grown on LB agar for 6 h. Bacterial lawn growth was resuspended in LB, diluted 1∶100 and re-plated onto LB agar containing kanamycin and rifampacin. A total of 7,000 kanamycin and rifampacin resistant colonies were streak-diluted on LB agar containing kanamycin and subsequently stored in LB containing 10% glycerol and kanamycin at −70°C until required. The sequences flanking the transposon insertions were amplified by Y-linker ligation PCR [22].

### Construction of *K. pneumoniae* deletion mutants

Knockout mutations, in which target genes were deleted by allelic exchange with a kanamycin resistance-encoding gene (*km*), were constructed in *K. pneumoniae* AJ218 using a “gene gorging” technique [79]. All primers used are listed in Table S1. ‘Donor’ plasmids carrying the desired mutation were constructed as follows. The *km* gene was amplified from pKD4 [80] using primers kanF and kanR. The resulting product included flanking fragment length polymorphism (FLP) recombinase target (FRT) sites to permit subsequent *km* excision [81]. Using *K. pneumoniae* AJ218 genomic DNA as the template, approximately 0.5 kb regions flanking the upstream and downstream sequence of the target gene were PCR amplified. The three fragments were joined together, with the *km* gene flanked by the upstream and downstream target gene sequences, in equimolar amounts using overlapping extension PCR [82]. The I*Sce*-I-flanked PCR products were cloned into pGEM-T Easy (Promega) to yield donor plasmids, which were then sequenced.

The ‘mutagenesis’ plasmid pACBSR carries genes encoding I-*Sce*I endonuclease and lambda Red recombinase under inducible control by L-arabinose [79]. Donor plasmids and pACBSR were transformed into electrocompetent *K. pneumoniae* AJ218 cells (0.1 cm gap-width cuvette; 200 ohms, 25 µF, 1.8 kV) and selected on LB agar containing kanamycin and chloramphenicol. A single co-transformant was inoculated into 1 mL LB containing 0.2% L-arabinose (Sigma-Aldrich, St. Louis, MO) and chloramphenicol and grown in a shaking incubator at 37°C for 16 h. Cell dilutions were grown on LB agar containing kanamycin, and resultant colonies were screened by colony PCR using primers flanking the targeted region and within the *km* gene. The loss of pACBSR was induced by 0.2% L-arabinose without selection. When required, the *km* gene was excised via the FRT sites using the FLP helper plasmid pCP20 [81].

### Creation of complementation constructs

Wild-type *K. pneumoniae* AJ218 genes were PCR amplified (Table S1), cloned into pGEM-T Easy and sequenced, followed by restriction enzyme digestion and insertion within the tetracycline resistance-encoding gene (*tet*) of pACYC184 via unique *Bam*HI/*Sal*I restriction sites. Apart from pMrkI, in which *mrkI* was transcribed from the *tet* gene promoter of pACYC184, all complement plasmids contained AJ218 genes that were transcribed from their native promoter, in the opposite orientation of the *tet* gene. Constructs were maintained in cells with chloramphenicol resistance selection.

### Construction of site-directed mutant constructs

Site-directed mutant alleles of *mrkH*, *mrkJ* and *yfiN* were constructed by overlapping-extension PCR [82] of wild-type *K. pneumoniae* AJ218 genomic DNA template using mutagenic oligonucleotides (Table S1). Overlapping primers were used together with the relevant upstream or downstream complementation primer. Amplified fragments were cloned into pGEM-T Easy and sequenced, followed by restriction enzyme digestion and insertion within the *tet* gene of pACYC184 or pBR322 via unique *Bam*HI/*Sal*I restriction sites.

### Static biofilm assays

Biofilm assays were performed according to O'Toole with minor modifications [21]. Transposon mutants were incubated statically overnight at room temperature in 96-well plates containing LB and kanamycin and subsequently diluted 1∶50 in 100 µL M63B1-GCAA minimal media (containing 1% glycerol and 0.3% casamino acids) in duplicate 96-well, flat bottom, non-tissue culture treated, polyvinyl chloride microtiter plates (Falcon; BD Biosciences, San Jose, CA). Wells containing growth medium alone were used as negative controls and the measurements of these wells were subtracted from the experimental measurements. Following 8 h static incubation at 37°C, planktonic bacteria were decanted and wells were washed twice with distilled water. Biofilms attached to well surfaces were stained for 15 min at room temperature with 125 µL of 0.1% (wt/vol) crystal violet solution (Sigma-Aldrich). The crystal violet solution was decanted and wells were subsequently washed twice with distilled water to remove traces of unbound dye. The bound dye was solubilized from adherent cells with 33% acetic acid and subsequently quantified by measuring the optical density at 595 nm. This assay was similarly applied to examine the formation of biofilms by gene deletion mutants, however strains were initially grown in 10 mL LB overnight in shaking conditions at 37°C and the length of the biofilm assay was 24 h before biofilms were stained and quantified. The data for each strain represent average values taken from four replicate wells performed in two independent experiments.

### Flow cell biofilm assays

For flow cell assays, biofilm culture media was a 1∶2,000 dilution of M63B1-GCAA culture media used for static biofilm assays. Biofilms were cultivated at 37°C in three-channel flow cells with individual channel dimensions of 1×4×40 mm (BioCentrum-DTU, Denmark). An ibidi cover slip (ibidi GmbH, Germany) was used as the substratum for biofilm growth. Flow cells were inoculated with overnight LB cultures diluted 1∶100 in warm culture media containing antibiotics as appropriate for plasmid-containing strains, and allowed to attach for 1 h at 37°C without flow. Flow was then commenced and maintained at 151 µL/min for 96 h. Biofilms were stained with 1.25 mM Syto64 (Invitrogen, Carlsbad, CA) to visualize cells and fixed with 4% paraformaldehyde.

### Microscopy and image analysis

Microscopic observations of biofilms and image acquisitions were performed utilizing confocal scanning laser microscopy (CSLM) on a Nikon A1 confocal microscope, using a Plan Apo 40×/1.42 oil objective. Images were reconstructed from Z-sections and rendered for 3D visualization using the IMARIS software package (Bitplane AG, Zurich, Switzerland). For statistical evaluation of biofilm structures, duplicate flow cells were prepared for each strain, and at least six image stacks (0.5 µm slices) per flow chamber were obtained for the mature biofilm at 96 h post-inoculation. The images were analyzed with the biofilm computer program COMSTAT [83].

### Detection of fimbriae expression

The presence of type 3 fimbriae was determined by mannose resistant hemagglutination (MRHA) assays [8]. Human erythrocytes (group A) were tanned by incubating equal volumes of 0.1% (wt/vol) tannic acid (Sigma-Aldrich) solution in saline and a 3% erythrocyte suspension in PBS for 15 min at 37°C. The erythrocytes were subsequently washed twice in PBS. Bacteria were grown overnight in shaking conditions in LB and subsequently washed twice and resuspended in PBS to approximately 1×10^10^ CFU/mL. A series of 2-fold dilutions of the bacterial suspension with or without 4% D-mannose (Sigma-Aldrich) was mixed with equal volumes (25 µL) of tanned erythrocytes in the depressions of porcelain tiles. The plates were rocked gently for 10 min at room temperature, after which the minimum bacterial density (CFU/mL) required to agglutinate erythrocytes was measured.

Type 3 fimbriae expression was also detected by immunoblot analysis using polyclonal rabbit antiserum prepared against purified MrkA. Whole cell lysates were prepared from 48 h M63B1-GCAA (containing 1% glycerol and 0.3% casamino acids) static cultures. Samples were separated by sodium dodecyl sulphate (SDS)-polyacrylamide gel electrophoresis (PAGE) and transferred to Hybond-C Extra nitrocellulose (Amersham Biosciences, Sweden) using a Trans-Blot SD Electrophoretic Transfer Cell (Bio-Rad Laboratories, Hercules, CA) at 12 V for 30 min. Goat anti-rabbit IgG-HRP (Invitrogen) was used as the secondary antibody at a concentration of 1∶3,000. Membranes were developed with TMB Membrane Peroxidase Substrate (KPL, Gaithersburg, MD). Loading was normalized by quantifying and comparing the relative differences of samples first separated by SDS-PAGE and stained with Coomassie-blue. The stained gel was scanned using a Kodak Digital Imaging System 4000MM (Eastman Kodak Company, Hemel Hempstead, England) and the intensity of a strongly expressed house-keeping gene product was measured to normalize the loading of samples in the subsequent immunoblot experiment.

### RNA preparation and RT-PCR conditions

Total RNA was extracted from *K. pneumoniae* AJ218 (grown to mid-log phase, OD600 = 0.8) using a FastRNA Pro Blue Kit (Qbiogene, Irvine, CA) and residual DNA was eliminated with a TURBO DNA-free Kit (Applied Biosystems/Ambion, Carlsbad, CA) according to the manufacturer's recommendations. First-strand cDNA synthesis was performed using 200 U SuperScript II Reverse Transcriptase (Invitrogen) using 3 µg total RNA and 20 pmole of the reverse primer: mrkI-R or mrkJ-R (Table S1). PCR of cDNA templates was performed using GoTaq Green Master Mix. Removal of contaminating DNA from the RNA sample was verified by PCR in the absence of reverse transcription. Reactions were separated by 1% (wt/vol) agarose gel electrophoresis.

### Quantitative RT-PCR

RNA was extracted from *K. pneumoniae* AJ218 strains and residual DNA eliminated, as stated above. cDNA was synthesized using 70 ng of random hexamers (Invitrogen) and Superscript II Reverse Transcription kit (Invitrogen), according to the manufacturer's guidelines. qPCR was performed using SYBR green (Quantace; SensiMix) and the primer pairs mrkA127F and mrkA265R and rpoD562F and rpoD677R (used at 0.125 µM each; Table S1). Cycling conditions were as follows: 95°C for 10 min followed by 35 cycles of 95°C for 30 sec, 55°C for 60 sec and 72°C for 15 sec. Primer efficiencies (E = 10^(−1/slope^) under these conditions were calculated following real-time PCR on 10-fold serially diluted genomic DNA, where the co-efficient for the standard curve was greater than 0.98. The efficiency-corrected, relative gene expression was determined using the Stratagene Mx3005 qPCR Thermocycler (Agilent Technologies, La Jolla, CA) whereby the expression of *mrkA* was normalised to the expression of *rpoD*. Primer specificity was determined by melting curve analysis and no Ct value was recorded for no template or no reverse transcriptase controls. All qPCR sample reactions were performed in triplicate.

### Construction of *lacZ* and *cat* transcriptional fusions

The *lacZ* and *cat* transcriptional fusions were constructed by PCR amplification of desirable DNA fragments using chromosomal DNA of *K. pneumoniae* AJ218 as template and the primers described in Table S1. PCR fragments were cloned into TOPO-TA (Invitrogen) or pGEM-T Easy cloning vectors and sequenced. The fragments were then each excised from the TOPO or pGEM derivatives and cloned into appropriate sites within plasmids pMU2385 and pKK232.8, to create *lacZ* and *cat* transcriptional fusions.

### Primer extension

Total cellular RNA was extracted (as stated above) from *E. coli* MC4100 strains containing pMrkH with either pMU2385 (control) or *mrkA-lacZ*-2. Cells were grown to mid-log phase (OD600 = 0.6). Primer Px1mrkARev (Table S1) was labeled at the 5′ end with both [γ-^32^P]ATP (Perkin Elmer, Waltham, MA) and T4 polynucleotide kinase (Promega) and subsequently co-precipitated with 5 µg of total RNA isolated from *E. coli* strain MC4100 containing pMrkH with either pMU2385 (control) or *mrkA-lacZ*-2. Hybridization was carried out at 45°C for 15 min in 10 mL of TE buffer containing 150 mM KCl. Primer extension reactions were started by the addition of 24 µL of extension solution (20 mM Tris HCl [pH 8.4], 10 mM MgCl_2_, 10 mM DTT, 2 mM dNTPs and 1 U/mL AMV Reverse Transcriptase) and were carried out at 42°C for 60 min. Samples were then precipitated and analyzed on a sequencing gel.

### β-galactosidase and CAT assays

β-galactosidase activity was assayed as described elsewhere [84]. Specific activity was expressed in units described therein. The data are the results of at least three independent assays. The CAT activity of mid-log-phase cultures, grown in LB, was assayed as described elsewhere [85]. The cells were disrupted by sonication, and cellular debris was removed by centrifugation before the assays were carried out. Each assay was performed at least three times. CAT activity was expressed as units per milligram of protein.

### Expression and purification of MrkH-8×His, MrkH(113R-A)-8×His and MrkJ-8×His

The coding regions of *mrkH*, *mrkH*:113R-A and *mrkJ* flanked by *Nde*I and *Bam*HI sites were PCR amplified using primer pairs mrkH(NdeI)11a and mrkH(BamHI)11a for *mrkH* and *mrkH*:113R-A, and mrkJ(NdeI)11a and mrkJ(BamHI)11a for *mrkJ* (Table S1) using pMrkH, pMrkH(113R-A) or pMrkJ as template, respectively. The amplified DNA fragments were cloned into TOPO-TA and sequenced. The *mrkH*, *mrkH*:113R-A and *mrkJ* fragments encoding the MrkH, MrkH:113R-A and MrkJ proteins with eight histidine residues tagged at the C-terminal end were then excised and cloned into the *Nde*I and *Bam*HI sites of pET11a (Novagen, Madison, WI) to form pET11a-mrkH-8His, pET11a-mrkH(113R-A)-8His and pET11a-mrkJ-8His. For over-expression of His-tagged proteins, *E. coli* expression strain BL21(DE3) containing pET11a-constructs was induced with 0.3 mM isopropyl-β-D-thiogalactopyranoside (IPTG) for 3 h at 20°C (for MrkH-8×His and MrkH(113R-A)-8×His) or at 16°C (for MrkJ-8×His). Over-expressed proteins were purified using Metal Affinity Chromatography.

### Electrophoretic mobility shift assay (EMSA)

Primer Px1mrkARev was labeled at the 5′ end with [γ-^32^P]ATP and T4 polynucleotide kinase. The DNA fragment containing the *mrkA* regulatory region was generated by PCR using ^32^P-labelled primers Px1mrkARev and mrk295F, with TOPO-TA carrying the *mrkA* regulatory region as template. Each end-labeled fragment was incubated with varying amounts of purified MrkH-8×His and MrkH(113R-A)-8×His protein with or without 200 µM c-di-GMP (enzymatically synthesized; for details see [86]) or varying amounts of purified MrkH-8×His with 200 µM GTP at 30°C for 20 min in the binding buffer (10 mM Tris HCl [pH 7.4], 50 mM KCl, 1 mM DTT, 100 µg/mL BSA and 5 ng/µL poly[dI-dC]). Glycerol was added to a final concentration of 6.5%. Samples were analyzed by electrophoresis on 5% native polyacrylamide gels (37.5∶1) containing 50 µM c-di-GMP. Electrophoresis was carried out at room temperature for approximately 8 h at 10 V/cm.

The binding affinities of PilZ-domain proteins with c-di-GMP are typically below 10 µM [50], [63], [87], [88]. To compensate for the loss of c-di-GMP through diffusion into the polyacrylamide gel and running buffer during electrophoresis, saturating concentrations of c-di-GMP (200 µM) were used to ensure that all MrkH molecules were ligand-bound. Saturating ligand concentrations have been used in other EMSA studies of protein interactions with c-di-GMP [63] and cAMP [89], [90].

### High-Performance Liquid Chromatography (HPLC)

The procedure for measuring phosphodiesterase activity has been described previously [86], [91]. Briefly, the purified MrkJ-8×His was incubated with 100 µM of c-di-GMP in 100 mM Tris buffer (pH 8.0) with 50 mM KCl and 25 mM MgCl_2_ at room temperature. Reactions were stopped by heating the reaction mixture at 95°C for 10 min. After the protein precipitate was removed by centrifugation at 135,000 rpm for 10 min, the supernatant was analyzed by HPLC. The formation of 5′-pGpG was monitored using an Agilent LC1200 system equipped with an XDB C18 column (4.6×150 mm) (mobile phase: 20 mM triethylammonium bicarbonate [pH 7.0], 10% methanol, 1 mL/min).

### Statistical analyses

All statistical analyses were performed using JMP 8.0 software (SAS Institute, Cary, NC). Data were log_10_/sqrt transformed for normality and analyzed by ANOVA with Tukey HSD post-hoc comparisons performed when necessary. Where normal distributions could not be met, non-parametric Van der Waerden tests were performed with Tukey HSD post-hoc comparisons when necessary. Where values were obtained as below the detection limit of an assay, the minimum detectable value minus 1 was assigned. *P*<0.05 was considered significant.

### Accession numbers

*K. pneumoniae* AJ218 gene sequences were deposited in GenBank under the accession numbers JF759917 (*mrkH*), JF759918 (*mrkI*), JF759919 (*mrkJ*), JF759920 (*yfiN*), and JF759921 (*mrkABCDF*).

## Supporting Information

Figure S1 **Biofilm formation by** ***K. pneumoniae*** **AJ218.** Biofilm formation by *K. pneumoniae* AJ218 wild-type and isogenic mutants strains +/- empty pACYC184 plasmids. Biofilm formation was determined using the static microtiter plate assay following incubation in M63B1-GCAA minimal media (supplemented with 1% glycerol and 0.3% casamino acids) for 24 h under static conditions. Results are expressed as a percentage of the biofilm produced by the wild-type AJ218 strain, which is set to 100%. All values represent the mean of four replicate sample wells for each strain performed in two independent experiments. The error bars represent the standard deviation.
(TIF)

Figure S2 **Type 3 fimbriae expression by** ***K. pneumoniae*** **AJ218.** Mannose resistant *Klebsiella*-like hemagglutination (MR/K HA) by *K. pneumoniae* AJ218 wild-type and isogenic mutant strains +/- empty pACYC184 plasmids using human erythrocytes. MR/K HA titer is expressed as the lowest concentration (CFU/mL) of bacteria causing a visible agglutination reaction. Values represent the mean of three independent experiments. The error bars represent the standard deviation.
(TIF)

Figure S3 **Coomassie-blue stained SDS-PAGE of over-expressed and purified MrkH-8×His (10 µg loaded).** The recombinant MrkH-8×His protein (used for EMSA studies) is labeled, which migrates at approximately 28 kDa.
(TIF)

Figure S4 **EMSA of the** ***mrkA*** **fragment.** The buffers and conditions used in the assay are as described in the Materials and Methods. The ^32^P-labelled PCR fragment containing the *mrkA* regulatory region was generated using primer pairs ^32^P-Px1mrkARev and mrk295F. The *mrkA* fragment was mixed with varying amounts of either the purified wild-type MrkH-8×His protein (from 0 to 500 nM) in the presence of 200 μM of GTP (left panel) or the purified mutant MrkH(113R-A)-8×His protein (from 0 to 500 nM) in the presence of 200 μM of c-di-GMP (right panel). Following incubation at 30°C for 20 min, the samples were analyzed on native polyacrylamide gels. The unbound DNA bands (F) are marked.
(TIF)

Figure S5 **Immunoblot of MrkH-8×His expression.** Samples were prepared by sonication followed by centrifugation and supernatant samples were separated by SDS-PAGE. Following transfer, the membrane was probed with anti-His antibody. Shown are *E. coli* MC4100 strains harboring pGMrkH-8His (wild-type) and pGMrkH(113R-A)-8His (mutant) preparations. *E. coli* MC4100 was used as the negative control. MrkH-8×His is labeled, which migrates at approximately 28 kDa.
(TIF)

Figure S6 **Coomassie-blue stained SDS-PAGE of over-expressed and purified MrkJ-8×His (10 µg loaded).** MrkJ-8×His protein (used for HPLC studies) is labeled, which migrates at approximately 29 kDa.
(TIF)

Table S1 Oligonucleotide primers used in this study.
(DOC)
