# Supplementary figures and images for: MrkH, a Novel c-di-GMP-Dependent Transcriptional Activator, Controls Klebsiella pneumoniae Biofilm Formation by Regulating Type 3 Fimbriae Expression
Source: PLoS Pathog. 2011 Aug 25;7(8):e1002204. doi: 10.1371/journal.ppat.1002204 (PMC3161979; doi:10.1371/journal.ppat.1002204)

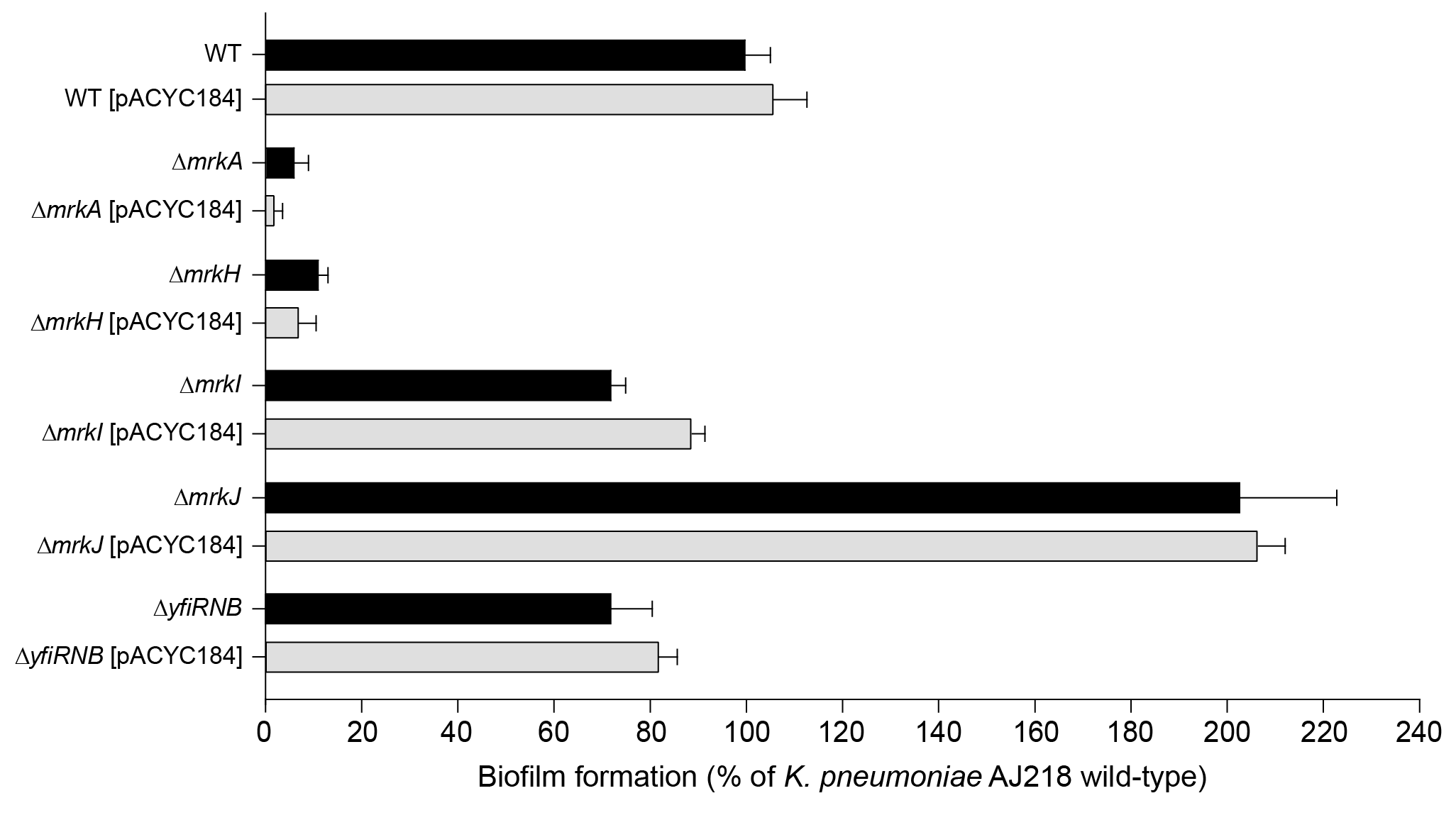

Supplement: Figure S1 — Biofilm formation by K. pneumoniae AJ218. Biofilm formation by K. pneumoniae AJ218 wild-type and isogenic mutants strains +/- empty pACYC184 plasmids. Biofilm formation was determined using the static microtiter plate assay following incubation in M63B1-GCAA minimal media (supplemented with 1% glycerol and 0.3% casamino acids) for 24 h under static conditions. Results are expressed as a percentage of the biofilm produced by the wild-type AJ218 strain, which is set to 100%. All values represent the mean of four replicate sample wells for each strain performed in two independent experiments. The error bars represent the standard deviation. (TIF) [file ppat.1002204.s001.tif]

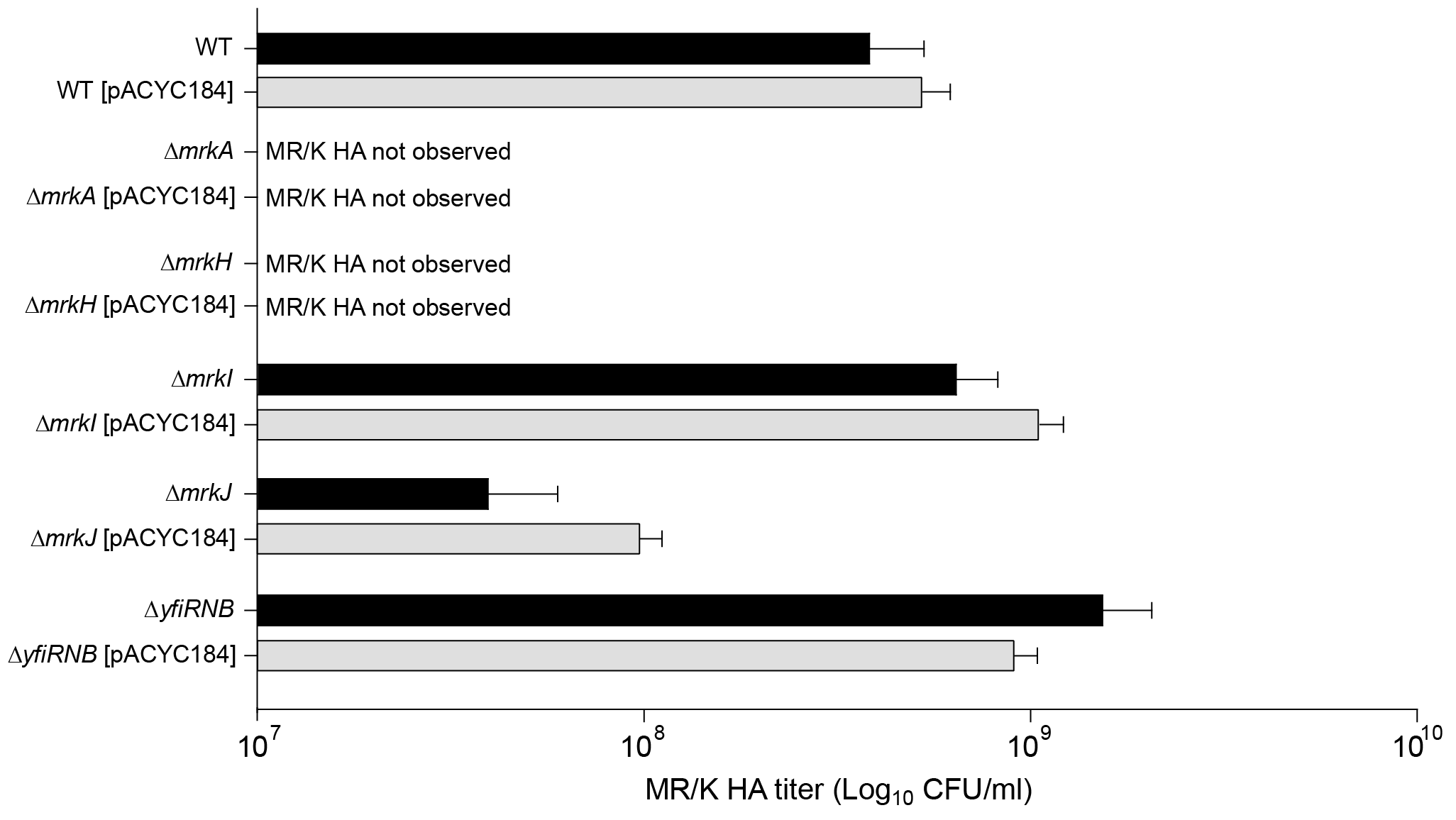

Supplement: Figure S2 — Type 3 fimbriae expression by K. pneumoniae AJ218. Mannose resistant Klebsiella-like hemagglutination (MR/K HA) by K. pneumoniae AJ218 wild-type and isogenic mutant strains +/- empty pACYC184 plasmids using human erythrocytes. MR/K HA titer is expressed as the lowest concentration (CFU/mL) of bacteria causing a visible agglutination reaction. Values represent the mean of three independent experiments. The error bars represent the standard deviation. (TIF) [file ppat.1002204.s002.tif]

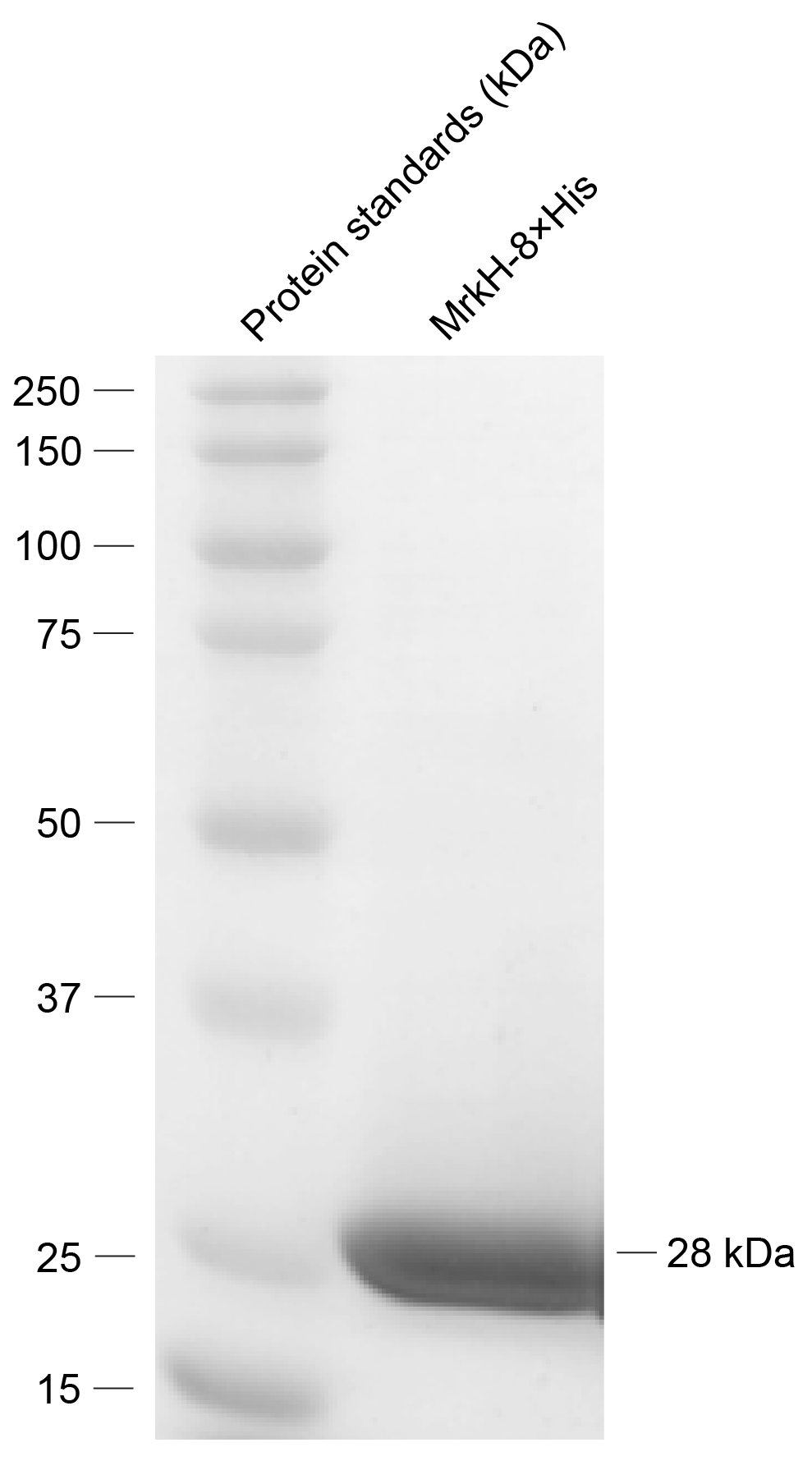

Supplement: Figure S3 — Coomassie-blue stained SDS-PAGE of over-expressed and purified MrkH-8×His (10 µg loaded). The recombinant MrkH-8×His protein (used for EMSA studies) is labeled, which migrates at approximately 28 kDa. (TIF) [file ppat.1002204.s003.tif]

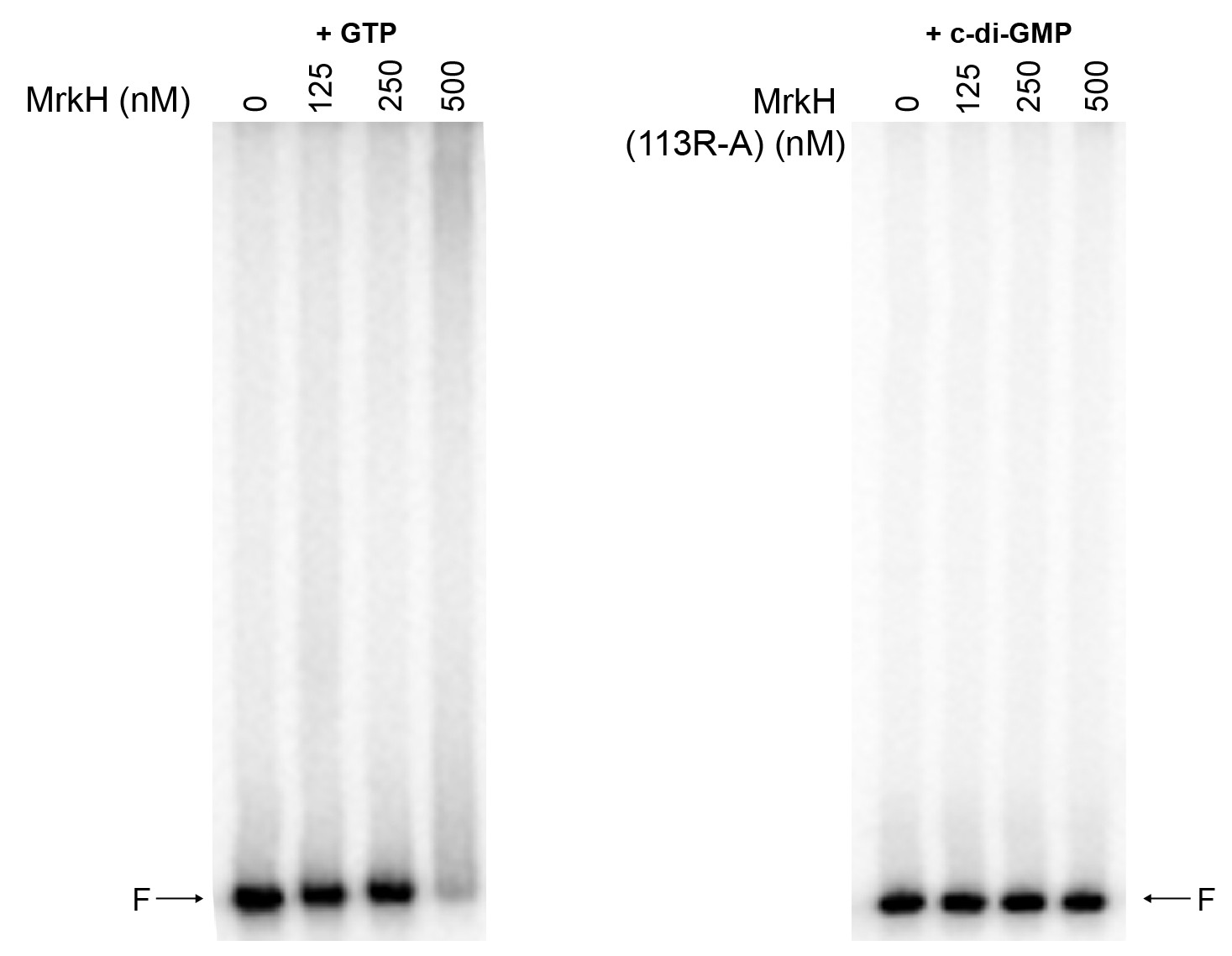

Supplement: Figure S4 — EMSA of the mrkA fragment. The buffers and conditions used in the assay are as described in the Materials and Methods. The 32P-labelled PCR fragment containing the mrkA regulatory region was generated using primer pairs 32P-Px1mrkARev and mrk295F. The mrkA fragment was mixed with varying amounts of either the purified wild-type MrkH-8×His protein (from 0 to 500 nM) in the presence of 200 μM of GTP (left panel) or the purified mutant MrkH(113R-A)-8×His protein (from 0 to 500 nM) in the presence of 200 μM of c-di-GMP (right panel). Following incubation at 30°C for 20 min, the samples were analyzed on native polyacrylamide gels. The unbound DNA bands (F) are marked. (TIF) [file ppat.1002204.s004.tif]

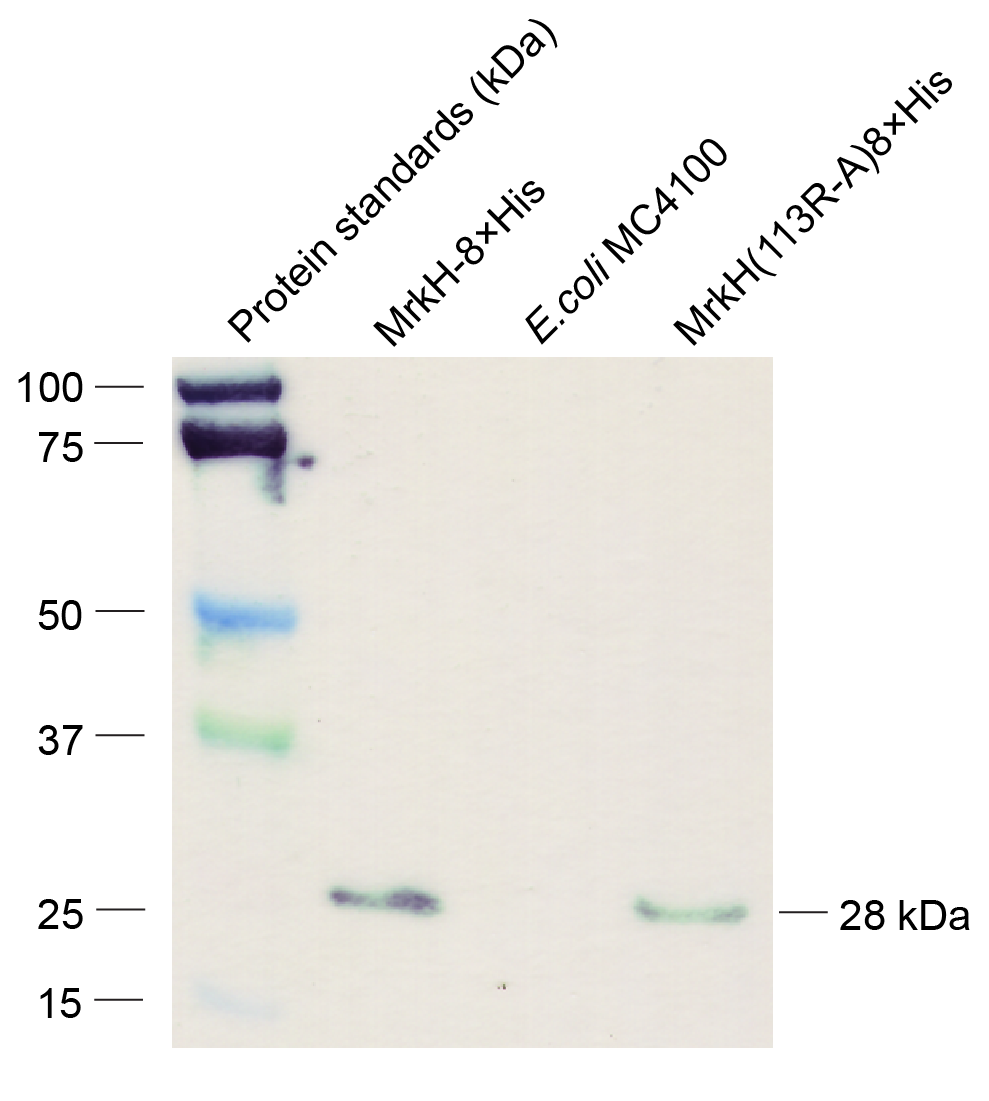

Supplement: Figure S5 — Immunoblot of MrkH-8×His expression. Samples were prepared by sonication followed by centrifugation and supernatant samples were separated by SDS-PAGE. Following transfer, the membrane was probed with anti-His antibody. Shown are E. coli MC4100 strains harboring pGMrkH-8His (wild-type) and pGMrkH(113R-A)-8His (mutant) preparations. E. coli MC4100 was used as the negative control. MrkH-8×His is labeled, which migrates at approximately 28 kDa. (TIF) [file ppat.1002204.s005.tif]

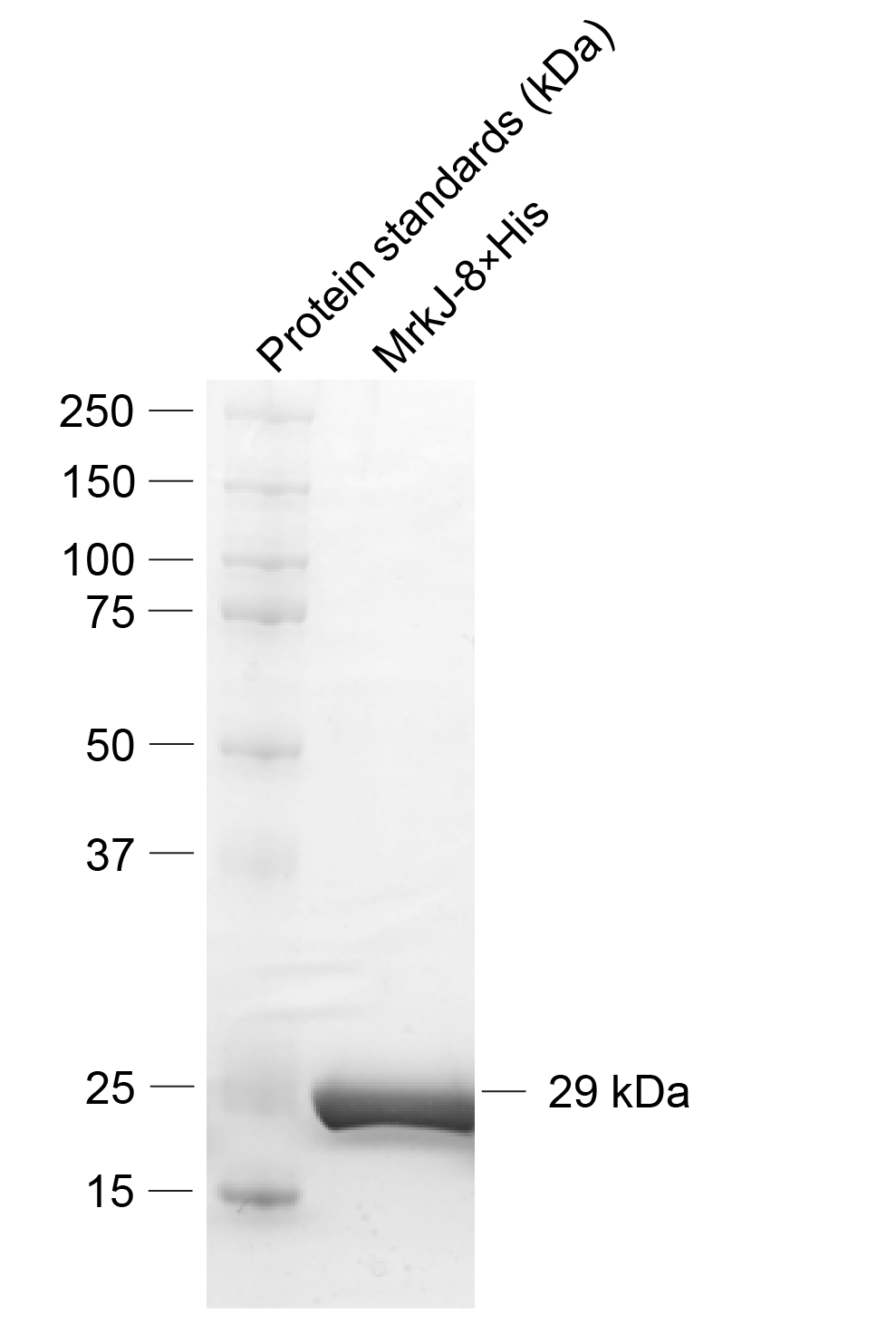

Supplement: Figure S6 — Coomassie-blue stained SDS-PAGE of over-expressed and purified MrkJ-8×His (10 µg loaded). MrkJ-8×His protein (used for HPLC studies) is labeled, which migrates at approximately 29 kDa. (TIF) [file ppat.1002204.s006.tif]
