# Supplementary material for: MrkH, a Novel c-di-GMP-Dependent Transcriptional Activator, Controls Klebsiella pneumoniae Biofilm Formation by Regulating Type 3 Fimbriae Expression
Source: PLoS Pathog. 2011 Aug 25;7(8):e1002204. doi: 10.1371/journal.ppat.1002204 (PMC3161979; doi:10.1371/journal.ppat.1002204)
Supplement: Table S1 — Oligonucleotide primers used in this study. (DOC) [file ppat.1002204.s007.doc]

**Table S1.** Oligonucleotide primers used in this study

| **Function** | **Primer name** | **Sequence (5’→3’)^a^** |
| --- | --- | --- |
| **Gene deletions** | |  |
| ***mrkA*** | mrkA(ISceI)F | TAGGGATAACAGGGTAATAATGCGCCTCATTCATGCTT |
|  | mrkA(ISceI)R | TAGGGATAACAGGGTAATAGCACGTTCTGGCCTTCATT |
|  | mrkAKanF | **CTAAGGAGGATATTCATATG**ACAGCTACGCGACTTACGAA |
|  | mrkAKanR | **GAAGCAGCTCCAGCCTACACA**TCCTTGTCAGAGTGAATTAC |
| ***mrkH*** | mrkH(ISceI)F | TAGGGATAACAGGGTAATGCGCCAAATATTCTGCTGAT |
|  | mrkH(ISceI)R | TAGGGATAACAGGGTAATTTGTTGGCAATGAGCAGTTC |
|  | mrkHKanF | **CTAAGGAGGATATTCATATG**AAGATACTGCTGGACCTGAT |
|  | mrkHKanR | **GAAGCAGCTCCAGCCTACACA**ATGCATCCCTTGTAAATAGT |
| ***mrkI*** | mrkI(ISceI)F | TAGGGATAACAGGGTAATGACAATAGCGGTGTCGATAA |
|  | mrkI(ISceI)R | TAGGGATAACAGGGTAATATCCTTCTGGCGCAACAGGT |
|  | mrkIKanF | **CTAAGGAGGATATTCATATG**ATCAGCGTATTGCCGCTCTC |
|  | mrkIKanR | **GAAGCAGCTCCAGCCTACACA**CTGTCCAAGGTTGTCAGATT |
| ***mrkJ*** | mrkJ(ISceI)F | TAGGGATAACAGGGTAATAATCGTCGATCTGCTGAATG |
|  | mrkJ(ISceI)R | TAGGGATAACAGGGTAATATGAAACCGCAGCCGTTTAC |
|  | mrkJKanF | **CTAAGGAGGATATTCATATG**GGCGGGAAGTCAGTATTGAA |
|  | mrkJKanR | **GAAGCAGCTCCAGCCTACACA**TATCCCTCTTCACGAGGTTA |
| ***yfiRNB*** | yfiRNB(ISceI)F | TAGGGATAACAGGGTAATGGAGCAAGGACCACATACCA |
|  | yfiRNB(ISceI)R | TAGGGATAACAGGGTAATCCACGATGCAGGTGGTGGAT |
|  | yfiRNBKanF | **CTAAGGAGGATATTCATATG**ATTTAGTCACCCGCGGCCTT |
|  | yfiRNBKanR | **GAAGCAGCTCCAGCCTACACA**CCGATGCCCGGCGAAGAATA |
| **Complementation constructs** | |  |
| ***mrkABCDF*** | mrk(SalI)F | TACGTCGACTAAGCCTGCTAACTATAACG |
|  | mrk(BamHI)R | TGGATCCACAACTGGTCGCCGATGATA |
| ***mrkH*** | mrkH(SalI)F | TACGTCGACACCGCTACTGGCACTATAGA |
|  | mrkH(BamHI)R | TGGATCCGCGGAGCGCATTCAGCAGAT |
| ***mrkI*** | mrkI(BamHI)F | TGGATCCAATGTCATTGTTATCACCCTGG |
|  | mrkI(SalI)R | TACGTCGACGGCTACCTGATGATTAATGG |
| ***mrkJ*** | mrkJ(SalI)F | TACGTCGACATCAGCGTATTGCCGCTCTC |
|  | mrkJ(BamHI)R | TGGATCCGGCGGCCTGTTCACCTATTA |
| ***yfiRNB*** | yfiRNB(SalI)F | TACGTCGACGCATGATTGCGATCCTGTGT |
|  | yfiRNB(BamHI)R | TGGATCCTCATTGCCGTTGACTACCTG |
| **RT-PCR** |  |  |
|  | mrkH-F | ACAACACCAGTATCATAACC |
|  | mrkI-R | CAATACGCTGATTCATAGTG |
|  | mrkI-F | CATTGGTCACTCAATCTATC |
|  | mrkJ-R | TCGACACCTTCAATAATCAC |
| **Y-linker ligation/PCR** | |  |
|  | Linker-1 | TTTCTGCTCGAATTCAAGCTTCTAACGATGTACGGGGACACATG |
|  | Linker-2 | TGTCCCCGTACATCGTTAGAACTACTCGTACCATCCACAT |
|  | Y-linker | CTGCTCGAATTCAAGCTTCT |
|  | Tn5 | GGCCAGATCTGATCAAGAGA |
| ***km* resistance gene** | |  |
|  | kanF | GTGTAGGCTGGAGCTGCTTC |
|  | kanR | CATATGAATATCCTCCTTAG |
| **Primer extension/EMSA** | |  |
|  | Px1mrkARev | GTGAATTCGCATAGAACCAGAAACATC |
| ***mrkA – lacZ*/*cat* fusions** | |  |
|  | mrk295F(BamHI) | cggatccgcgttttcatctatcaatggctg |
|  | mrk109R(HindIII) | caagctttaccgaagaaattaacctggccg |
|  | mrk795F(BamHI) | cggatccgaaagagaccagggagagcatac |
|  | mrkAdel(HindIII) | GAAGCTTGTTCGCTGGTGCTATCGGCG |
|  | mrkAdel(BamHI) | AGGATCCGATGGTTATCTGTTATATAACTTAATGAAACGTG |
| **8×His constructs** | |  |
| *mrkH*-8×His | mrkH(NdeI)11a | ACATATGACAGAGGGAACGATAAAGACC |
|  | mrkH(BamHI)11a | AGGATCCTTAGTGGTGGTGGTGGTGGTGGTGGTGGATTCTCTTTTTGCGCTTGGCTTC |
| *mrkJ*-8×His | mrkJ(NdeI)11a | ACATATGAACACTAAAATATTCGAAGACAACATTTTATCTCG |
|  | mrkJ(BamHI)11a | AGGATCCTTAGTGGTGGTGGTGGTGGTGGTGGTGCATGGCAATATCATCGGCGACCAG |
| **Overlapping site-directed mutagenesis** | |  |
| *mrkH* (113R-A) | mrkH(113R-A)F | GCGTCGCCGCGATCCCGCCTTTCGTTTACGCCATG |
|  | mrkH(113R-A)R | CATGGCGTAAACGAAAGGCGGGATCGCGGCGACGC |
| *mrkJ* (36ECL-AAA) | mrkJ(36ECL-AAA)F | CTGGTCGCGGTGGCTGCTGCAAGCCGCTTTGATAATC |
|  | mrkJ(36ECL-AAA)R | GATTATCAAAGCGGCTTGCAGCAGCCACCGCGACCAG |
| *yfiN* (328DEF-AAA) | yfiN(328DEF-AAA)F | CGTCTTGGCGGCGCAGCAGCTGCCGTTCTGCTG |
|  | yfiN(328DEF-AAA)R | CAGCAGAACGGCAGCTGCTGCGCCGCCAAGACG |
| **Quantitative RT-PCR** | |  |
| *mrkA* | mrkA127F | AGCGATGCGAACGTTTACCTGTCTC |
|  | mrkA265R | CGTCATCCTGTTTAGTGCCATCAGC |
| *rpoD* | rpoD562F | gaagagatggatgacgacgaagacg |
|  | rpoD677R | gtacgcagctcggcgaatttctcac |

^a^Restriction endonuclease recognition sites are underlined. Kanamycin resistance-encoding gene-specific sequences in gene deletion primers are in boldface.
